# Orbital tumours and tumour-like lesions: exploring the armamentarium of multiparametric imaging

**DOI:** 10.1007/s13244-015-0443-8

**Published:** 2015-10-31

**Authors:** Bela S. Purohit, Maria Isabel Vargas, Angeliki Ailianou, Laura Merlini, Pierre-Alexandre Poletti, Alexandra Platon, Bénédicte M. Delattre, Olivier Rager, Karim Burkhardt, Minerva Becker

**Affiliations:** Department of Radiology, Geneva University Hospital and University of Geneva, Rue, Gabrielle-Perret-Gentil 4, 1211 Geneva 14 Switzerland; Department of Neuroradiology, Geneva University Hospital and University of Geneva, Rue, Gabrielle-Perret-Gentil 4, 1211 Geneva 14 Switzerland; Department of Nuclear Medicine, Geneva University Hospital and University of Geneva, Rue, Gabrielle-Perret-Gentil 4, 1211 Geneva 14 Switzerland; Department of Clinical Pathology, Geneva University Hospital and University of Geneva, Rue, Gabrielle-Perret-Gentil 4, 1211 Geneva 14 Switzerland

**Keywords:** Orbit tumours, Magnetic resonance imaging (MRI), Diffusion weighted imaging (DWI), Positron emission tomography CT (PET CT), Positron emission tomography MRI (MRI PET)

## Abstract

Although the orbit is a small anatomical space, the wide range of structures present within it are often the site of origin of various tumours and tumour-like conditions, both in adults and children. Cross-sectional imaging is mandatory for the detection, characterization, and mapping of these lesions. This review focuses on multiparametric imaging of orbital tumours. Each tumour is reviewed in relation to its clinical presentation, compartmental location, imaging characteristics, and its histological features. We herein describe orbital tumours as lesions of the globe (retinoblastoma, uveal melanoma), optic nerve sheath complex (meningioma, optic nerve glioma), conal-intraconal compartment (hemangioma), extraconal compartment (dermoid/epidermoid, lacrimal gland tumours, lymphoma, rhabdomysarcoma), and bone and sinus compartment (fibrous dysplasia). Lesions without any typical compartmental localization and those with multi-compartment involvement (veno-lymphatic malformation, plexiform neurofibroma, idiopathic orbital pseudotumour, IgG4 related disease, metastases) are also reviewed. We discuss the role of advanced imaging techniques, such as MR diffusion-weighted imaging (DWI), diffusion tensor imaging, fluoro-2-deoxy-D-glucose positron emission tomography CT (FDG-PET CT), and positron emission tomography MRI (MRI PET) as problem-solving tools in the evaluation of those orbital masses that present with non-specific morphologic imaging findings.

*Main messages/Teaching points*

• *A compartment-based approach is essential for the diagnosis of orbital tumours.*

• *CT and MRI play a key role in the work-up of orbital tumours.*

• *DWI, PET CT, and MRI PET are complementary tools to solve diagnostic dilemmas.*

• *Awareness of salient imaging pearls and diagnostic pitfalls avoids interpretation errors.*

## Introduction

The orbit is a small anatomical space with a wide range of important structures within. Tumours and tumour-like lesions often arise from these orbital contents and are a common indication for the radiological evaluation of the orbit in both adults and children. Cross-sectional imaging plays a vital role in the diagnosis and management of these lesions. Knowledge of the clinical presentation and patient’s age helps to limit the differential diagnosis and to determine the appropriate imaging modality. Certain orbital pathologies such as retinoblastoma and rhabdomyosarcoma (RMS) are typically found in children, whereas malignant uveal melanoma, lymphoma, and inflammatory orbital pseudotumour (IOP) are seen in adults. Clinical symptoms such as extraocular muscle palsies, diplopia, visual impairment, exophthalmos, and eye pain can serve as useful pointers to the likely pathology. However, a biopsy may be needed to provide tissue diagnosis [1–4].

The purpose of this review is to discuss the clinical, imaging, and histopathological features of commonly encountered orbital tumours and tumour-like conditions. We highlight the role of cross-sectional imaging in the evaluation of indeterminate orbital masses with emphasis on advanced imaging techniques such as MR diffusion weighted imaging (DWI), diffusion tensor imaging (DTI), fluoro-2-deoxy-D-glucose (FDG)-positron emission tomography CT (PET CT), and positron emission tomography MRI (PET MRI or MRI PET, the European Congress of Radiology uses MRI PET as the abbreviation to emphasize its imaging value). We discuss their impact on the differential diagnosis of those lesions, where conventional morphologic cross-sectional imaging is non-specific.

## Imaging techniques

Evaluation of suspected orbital masses can be done with magnetic resonance imaging (MRI), computed tomography (CT), ultrasound (US), fluoro-2-deoxy-D-glucose positron emission tomography/CT (FDG-PET CT), and, recently, MRI PET. Most investigators agree that MRI is the imaging modality of choice, whereas CT should be reserved for cases with suspected bony pathology and whenever MRI cannot be performed. As shown by several investigators, high resolution MRI (HR MRI) shows superior soft tissue contrast than CT and allows more accurate depiction of the different orbital compartments [1–3]. MRI examinations can either be performed on a 1.5 T or on a 3 T scanner using a combination of head and surface coils. The standard MRI protocol in most institutions consists of axial SE T1W and TSE T2W sequences, coronal STIR sequences, and axial and coronal SE T1W sequences with fat saturation obtained after intravenous administration of gadolinium chelates. The recommended slice thickness is 2–3 mm with a 512 × 512 matrix. 3D FT heavily T2W sequences (CISS, FIESTA, or DRIVE) and 3DFT T1W sequences with 0.6-1 mm thin slices (VIBE, THRIVE) after intravenous contrast material administration are additionally used by some investigators including ourselves, as these volumetric data sets allow multiplanar reconstructions in any given plane, thereby facilitating the evaluation of subtle findings.

In patients with suspected orbital masses, CT is the modality of choice for evaluating calcifications and osseous orbital lesions and in patients with possible metallic foreign bodies [1–3]. CT examinations are usually performed using 0.6–1 mm thin slices after intravenous injection of iodinated contrast material. Thin-slice HR multidetector CT provides quick volumetric acquisitions and precise depiction of the globe, optic nerve, intraconal, and extraconal spaces. Standard coronal and sagittal reconstructions are routinely obtained with bone and soft tissue settings.

Because of the superficial location of the globe and its cystic nature, ultrasound (US) with Doppler allows accurate depiction of a variety of pathologic conditions of the globe and orbit, and enables non-invasive and cost-effective follow-up. The technique is well tolerated, easy to perform, and has a high accuracy for the characterization of vascular lesions in skilled hands.

### Role of advanced imaging

In recent years, advanced imaging techniques such as DWI, DTI, FDG-PET CT, and MRI PET are being increasingly used in the pre-therapeutic work-up and post-therapeutic monitoring of patients with head and neck tumours [5–15]. These modalities are also making significant inroads in ophthalmologic imaging. The physiological and functional information obtained by these techniques can be used to complement morphological findings obtained from conventional imaging, thereby aiding non-invasive tissue characterization [4, 8–23]. Although DWI sequences are widely used to assess primary or recurrent head and neck tumours, their applications in the orbit are still limited in clinical routine due to geometric distortion caused by the adjacent bone, air, and soft tissue interfaces. Nevertheless, the development of newer, robust single shot echo-planar DWI or multishot echo-planar DWI (such as the RESOLVE sequence) holds promise for the evaluation of orbital tumours and optic nerve pathology. DWI produces contrast on the basis of diffusivity of water molecules in different tissues and can thus be a source of information for assessing pathological tissue. Echo-planar DWI can help to differentiate between benign and malignant orbital masses based on apparent diffusion coefficient (ADC) values. DWI is more useful in characterizing infiltrative orbital masses, which appear hypointense on T2W images as compared to well-defined hyperintense lesions. Malignant masses especially orbital lymphomas show visually and quantitatively lower ADC values as compared to benign masses. Restricted diffusion is attributed to the higher cellularity and higher nuclear: cytoplasmic ratios in malignant lesions. Sepahdari et al have reported that an ADC value of 1.0 – 1.15 × 10^−3^ mm^2^/s represents an optimal threshold for predicting malignancy with a reported sensitivity of 95 %, specificity of 91 % and accuracy of 93 % [9, 10]. Visual assessment of benign and malignant lesions may not reveal significant differences on just DW images because of the T2-shine through effect. However, ADC maps are only affected by diffusivity changes. DWI can help to localize malignant masses in a background of non-specific inflammation and guide biopsy or intervention [9–13].

DTI is a novel MRI technique, which allows anatomic mapping and quantitative characterization of white matter architecture by measuring molecular diffusion [24]. DTI tractography is a computational procedure, which reconstructs white matter tracts in 3D-space based on their anisotropy characteristics. Tractography is used to provide a roadmap for the pre-surgical assessment of white matter tracts involved by tumours. Advances in MR data acquisition and post-processing now permit high-resolution DTI of cranial nerves in a clinical setting. The antero-posterior orientation and fairly large size of the optic nerve and tracts provide favourable parameters for tractography. DTI can be used to map optic nerve fibres when involved by tumourous conditions such as gliomas or meningiomas. Depending on whether the tumour displaces or infiltrates the nerve fibres, appropriate function-saving nerve surgery can be planned [3, 14–16]. However, DTI images of the optic nerve may show severe geometric distortion, in particular in the region of the orbital apex, and fibre tracking of the optic nerve is still part of ongoing clinical research protocols.

FDG-PET CT has recently emerged as a problem-solving tool in ophthalmologic oncology. It provides functional information regarding tumour metabolism based on FDG uptake. The level of FDG uptake depends upon tumour type and grade, size, surrounding metabolic activity, and blood glucose levels. For most primary orbital lesions, PET CT does not seem to provide any significant advantage over clinical evaluation and CT/MR imaging, other than detecting distant and metastatic lesions missed by conventional imaging. However, PET CT is now part of standard protocol in the initial staging work-up and post treatment assessment of orbital/ocular adnexal lymphomas. PET/CT also seems appropriate for metastatic tumours to the orbit and ocular adnexae from other primary sites. In these cases, one single comprehensive study may be preferable to performing multiple CT scans with contrast. The potential pitfalls in FDG-PET CT imaging of the orbit include background physiological uptake in extra-ocular muscles, poor uptake in very small lesions, and the likelihood of missing subtle uptake due to being at the edge of the field of view in standard skull to upper thigh PET CT scans [21]. Comparing standardized uptake values (SUV) between malignant and benign lesions and lesion to surrounding tissue helps in increasing diagnostic accuracy [17–21].

MRI PET leverages the superior soft-tissue contrast and functional sequences of MRI with the molecular information of PET in a single hybrid imaging technique. MRI PET is still a clinical research tool, however, recent publications have shown that MRI PET has an equivalent performance with respect to lesion detection as PET CT in the evaluation of head-neck cancers [7, 23]. Although large-scale data and studies on the role of MRI PET in orbital conditions are still awaited, it can be a considered as a potential future tool in refining orbital imaging. Paediatric and pregnant patients may benefit from MRI PET because of reduced radiation burden as compared to PET CT. MRI PET may play a valuable role in the evaluation of paediatric lymphomas, neuroblastomas. and soft tissue sarcomas [22, 23].

## Compartment-based approach to orbital masses

**Fig. 1:**
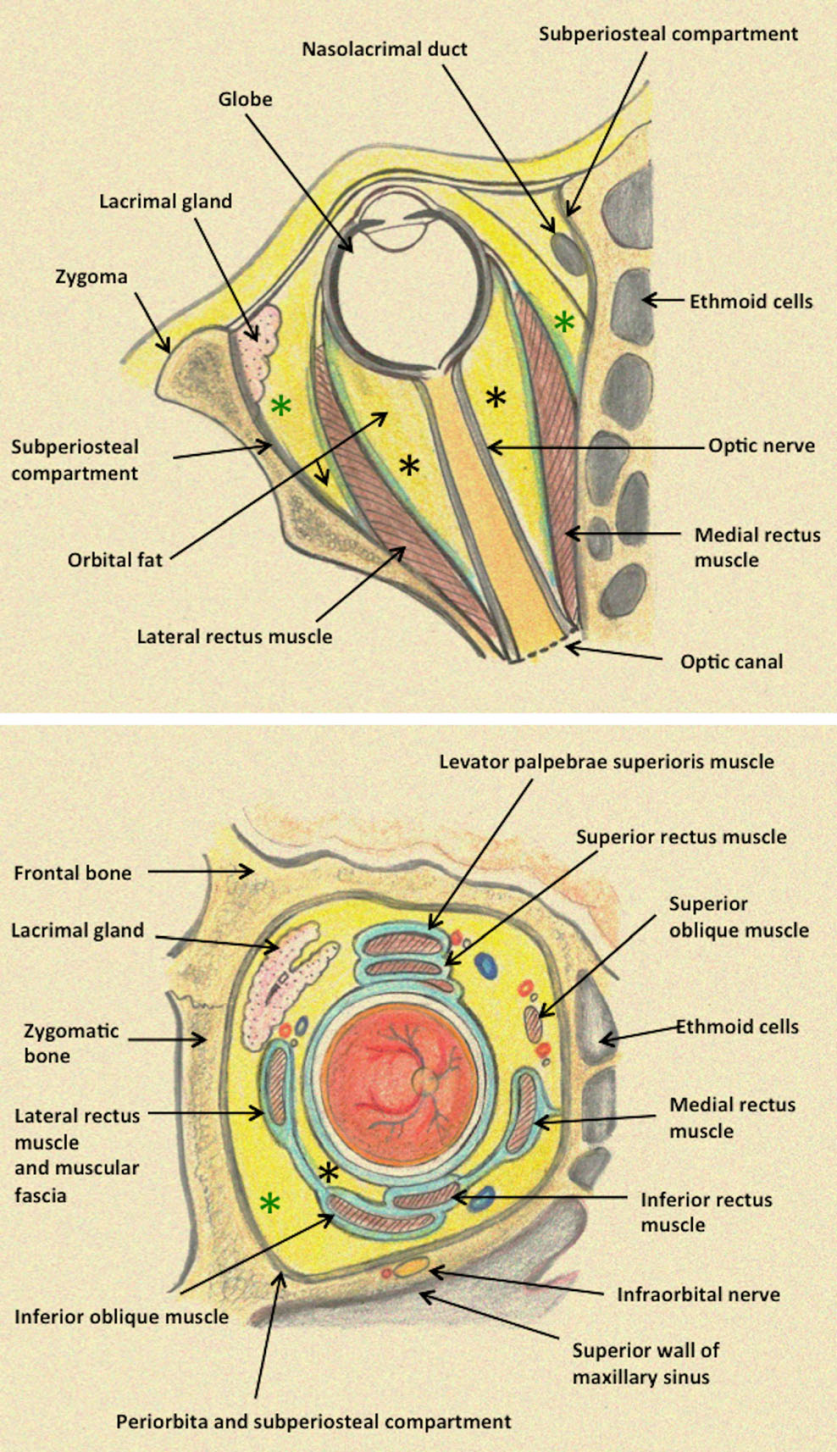
Schematic illustration of the orbital contents and compartments. Axial and coronal view of the right orbit. Black asterisks indicate the intraconal compartment. Green asterisks indicate the extraconal compartment

The orbit can be anatomically divided into some well-defined compartments (Fig. 1). The muscle cone comprising the four rectus muscles divides the orbit into the intraconal and extraconal compartments. The intraconal compartment contains the globe, the optic nerve-sheath complex, orbital vessels. and nerves (Fig. 1). The extraconal compartment consists of the bony orbital walls, fat. and the lacrimal gland. The orbital septum and lid form the anterior or preseptal compartment. Localizing orbital lesions to these specific compartments helps to simplify the diagnostic approach and narrow down the list of differentials [1].

In this review, we will describe tumours of the globe and optic nerve first, followed by the conal-intraconal tumours, extraconal tumours, and finally tumours of the bones/sinuses (Table 1). Examples of multi-compartmental masses will also be discussed (Table 1).

**Table 1:** Overview of orbital tumours and tumour-like lesions using a compartment based approach. Asterisks indicate possible, however, rare disease manifestations in the respective compartment

Intraconal and conal masses			Extraconal masses			Multi-compartment masses
Globe	Optic nerve sheath complex	Muscle cone and retrobulbar fat	Orbital appendages (lacrimal gland and sac)	Other extraconal structures, bones, and sinuses	Eyelid	
Retinoblastoma	Optic nerve glioma	Cavernous hemangioma	Epithelial lacrimal gland tumours (benign mixed tumour, adenoid cystic carcinoma, adenocarcinoma, undifferentiated carcinoma)	Dermoid/epidermoid	Eyelid tumours (squamous cell carcinoma, basal cell carcinoma, melanoma, lymphoma)	Vascular malformations
Hemangioblastoma	Optic nerve sheath meningoma	Pseudotumour	Lymphoma, leukemia	Rhabdomyosarcoma		Lymphoma
Uveal melanoma	Lymphoma*, leukemia*	Schwannoma*of cranial nerves III, IV, VI	Sarcoidosis	Schwannoma and perineural spread V1 and V2		Rhabdomyosarcoma
Chorioid metastases			Pseudotumour	Fibrous dysplasia, osteoma, metastases, myeloma		Plexiform neurofibroma
						Metastases
						Pseudotumour
						Ig-G4 related disease

### Globe

#### Retinoblastoma

Retinoblastoma is a malignant, primary retinal neoplasm and the most common intraocular tumour of childhood. About 90 % of cases occur under 5 years of age. Retinoblastoma can occur in a hereditary form (40 %) or in a sporadic form (60 %). In both cases, patients have mutations of the retinoblastoma tumour suppressor gene on chromosome 13q14 [1, 3, 4, 25, 26]. Hereditary retinoblastoma is associated with early onset disease; screening ultrasound (US) and MRI have been recently recommended for the management of fetuses at high risk of developing this tumour [27]. The higher incidence of retinoblastoma seen in developing countries has been partly attributed to the presence of human papilloma virus in tumour tissue [28]. About 50 % of cases present with leukocoria and 40 % show bilateral disease. Although the initial diagnosis is based on ophthalmoscopy and US, cross-sectional imaging is mandatory to assess disease extent and prognosis. Extra-ocular extension occurs in less than 10 % cases and portends poor prognosis. When a pinealoblastoma is associated with bilateral retinoblastomas, the term trilateral retinoblastoma is applied. Low-grade intra-ocular retinoblastomas are treated with chemotherapy while advanced tumours require enucleation and/or radiotherapy (RT) [1, 3, 4, 25, 26].

**Fig. 2:**
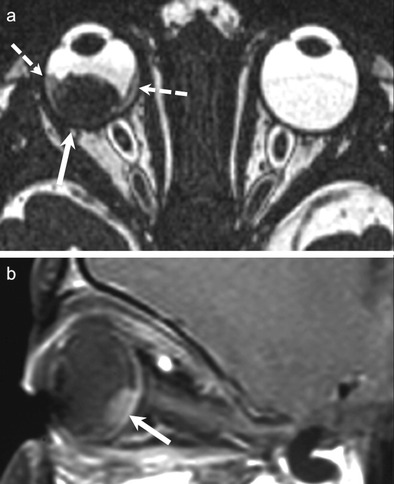
3-year-old boy with right-sided retinoblastoma. **a.** Axial 3D T2W image of the orbits shows a well-circumscribed retinal mass (solid arrow), which appears very hypointense as compared to the surrounding bright vitreous. Associated retinal detachment/haemorrhage (dashed arrows) appears moderately hypointense. **c.** Sagittal contrast-enhanced FS T1W image of the same patient shows avid tumour enhancement (arrow). The tumour is limited to the globe. No other lesions were seen intracranially

Retinoblastoma arises from precursor cells of the retinal neuroepithelium. Histologically, undifferentiated areas of the tumour show characteristic "small blue cells" with scant cytoplasm and large hyperchromatic nuclei. Differentiated structures called Flexner-Wintersteiner rosettes are commonly seen. Because of rapid growth of the tumour, necrosis and calcifications are common; these features account for the characteristic radiologic appearances on CT and MRI [25, 26]. Non-contrast-enhanced CT (NECT) demonstrates intra-tumoural calcifications in about 90 % cases. Marked enhancement of the tumour is seen on contrast-enhanced CT (CECT). MRI of the orbits and brain is usually performed together to determine extraocular and intracranial extension as well as to rule out an associated pinealoblastoma. The tumour is slightly hyperintense on T1W images, hypointense on T2W images, and shows marked post contrast enhancement on contrast-enhanced MRI (CEMRI) (Fig. 2) [1, 4, 25, 29]. Although CT is superior in calcium detection, MRI may allow equally accurate identification of calcifications by revealing spots of low signal intensity on very thin T2 TSE and T2* weighted sequences [30]. Retinoblastoma may exhibit variable hyperintensities on DWI resulting in varying values on ADC maps, depending on cellularity and histology. De Graaf et al have reported statistically significant differences in ADC values in viable enhancing tumour tissue (1.03 × 10 ^−3^ mm ^2^/s) as compared to non-viable necrotic tissue (1.47 × 10 ^−3^ mm ^2^/s). Thus ADC values can be used for monitoring treatment response in retinoblastoma [31].

Other diseases causing leukocoria can be differentiated from retinoblastoma based on clinical presentation and imaging characteristics. Coat’s disease, persistent primary hyperplastic vitreous and toxocariasis lack calcifications. Retinopathy of prematurity rarely shows calcifications, it may be bilateral and history of prematurity is usually elicited [4, 25, 26].

Unlike in adult cancers, the role of PET CT in paediatric patients is still not widely established. As intraocular retinoblastomas may not be FDG avid, it appears that PET CT does not have a role in their evaluation. Nevertheless, it has been suggested that increased optic nerve uptake on baseline PET CT may be a predictor of lower event free survival and lower overall survival as compared to patients without optic nerve FDG uptake [32].

#### Malignant uveal melanoma

Malignant uveal melanoma is the most common primary intraocular tumour in adults. The incidence is highest amongst Caucasians with peak incidence at 53 years. Malignant melanoma arises from the choroid in 85 % of cases. The diagnosis is usually made on ophthalmoscopy and US; however, cross-sectional imaging is necessary in case of opaque lens or significant subretinal effusion. Poor prognostic factors include age > 60 years, large tumour size, anterior uveal location and heavy tumour pigmentation. Photocoagulation, radiotherapy (RT), and globe sparing surgery are treatment options for smaller tumours while enucleation is necessary for larger tumours. Systemic metastases to the liver, lungs, and brain are often the cause of mortality [18, 26, 33].

The current WHO classification of choroidal tumours consists of three histological types, i.e. mixed, epitheloid, and necrotic melanomas. Spindle cell A tumours are now classified as benign choroidal nevi. The mixed type includes spindle B and epitheloid cells. The prognosis of spindle cell tumours is better as compared to mixed or purely epitheloid melanomas. Metastatic potential is determined by maximum tumour dimensions, number of epithelioid cells, vascular patterns, and nucleolar size and activity [26, 33, 34].

**Fig. 3:**
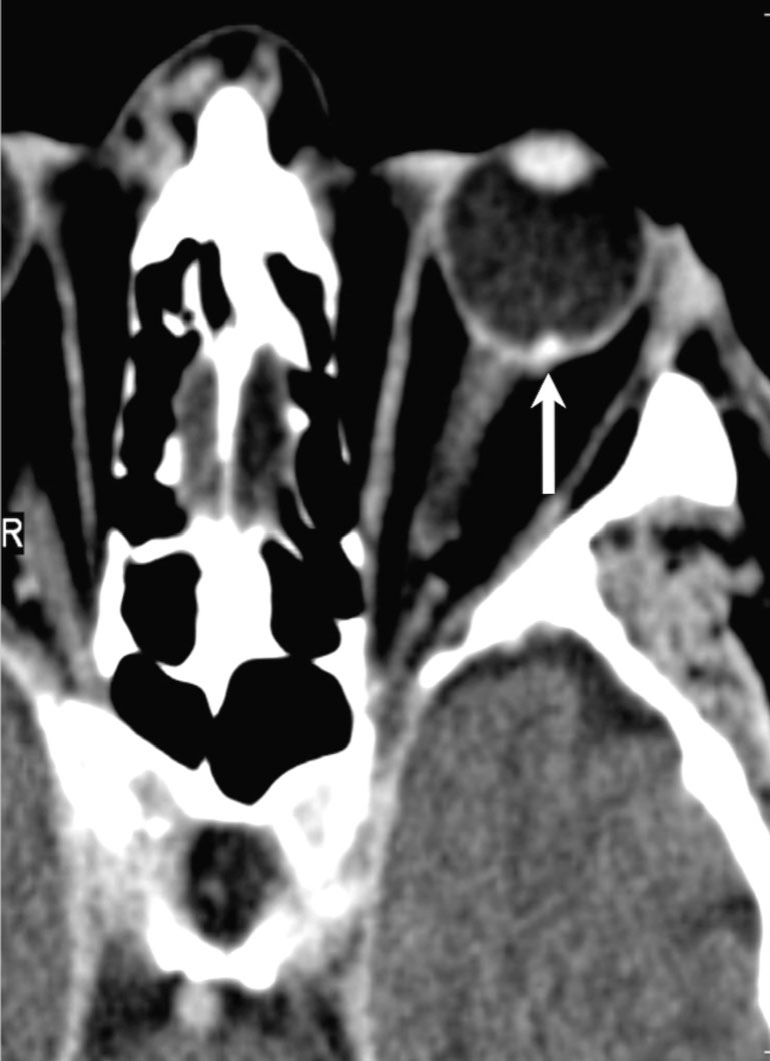
50-year-old male patient diagnosed with a left-sided uveal melanoma on ophthalmoscopy. **a.** Axial contrast-enhanced CT image shows a tiny, avidly enhancing nodule (arrow) along the choroid

NECT shows a well-circumscribed, hyperdense, mushroom-shaped tumour with a broad choroidal base. Calcification is rare. CECT and CEMRI show marked post contrast enhancement (Fig. 3). The melanin content causes characteristic hyperintense signal on T1W and hypointense signal on T2W MR images. Thin-section MRI enables accurate prediction of the degree of melanomatous pigmentation based on quantitative evaluation of T1W images. More pronounced pigmentation is associated with poorer prognosis. Fat-saturated, high-resolution CEMRI is ideal for demonstrating small enhancing tumours, associated retinal detachment as well as extraocular disease. Imaging differentials include choroidal hemangioma, choroidal detachment, and uveal metastases [33]. False positive or false negative assessments for ocular melanoma and other ocular tumours may be caused by retinal detachment and highly myopic patients. In highly myopic patients, the globe can be elongated and the resulting chemical shift artefact can lead to asymmetric thickening of the outline of the globe mimicking the presence of a tumour while, on the contrary, retinal detachment may mask small tumours. Erb-Eigner et al have reported that ocular melanomas show marked restricted diffusion with a mean ADC value of 0.891 × 10^−3^mm^2^/s. Also, the mean ADC of ocular melanoma appears to be statistically significant from the mean ADC of retinal detachment [35] thereby facilitating diagnosis.

FDG-PET CT has the potential to provide substantial benefit in the surveillance of uveal melanomas. Reddy et al found that PET CT was able to detect metabolic activity of choroidal melanomas, especially T3 and larger lesions. A positive correlation was found between SUV and the size of the melanoma. However, PET CT is unable to differentiate between small melanomas and suspicious choroidal nevi [36]. Whole body PET CT has been shown to play a valuable role in detecting regional and distant metastases from choroidal melanoma. Kurli et al have reported 100 % sensitivity and specificity of PET CT in the identification of hepatic metastases of choroidal melanomas [37].

### Optic nerve-sheath complex

#### Optic nerve glioma (ONG)

**Fig. 4:**
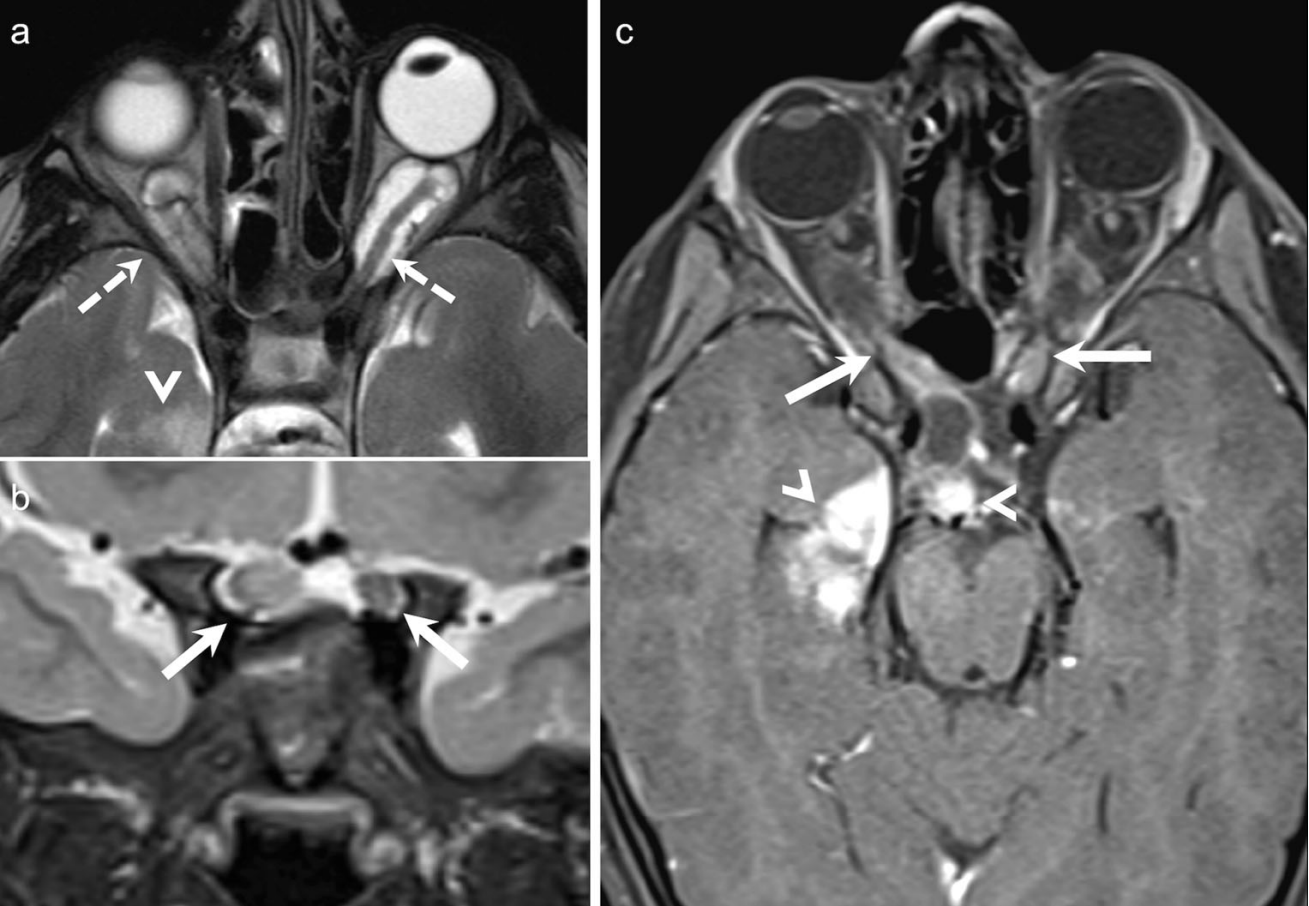
5-year-old boy with NF-I. **a.** Axial T2W MR image of the orbits shows bilateral ONG (dashed arrows) causing fusiform enlargement and kinking of the optic nerves. A focal T2- hyperintense lesion (arrowhead) is seen in the right mesial temporal lobe. **b.** Coronal T2WMR image of the same patient shows extension of the bilateral ONG along the intracranial segments of the optic nerves (arrows). **c**. Axial contrast-enhanced FS TIW MR image of the same patient shows prominent enhancement in the intracanalicular and intracranial segments of bilateral ONG (arrows). There is also avid enhancement in the suprasellar/chiasmatic region (arrowhead) and in the right mesial temporal lesion (arrowhead) in keeping with tumour extension along the optic chiasm and right optic radiation

ONG is the most common primary optic nerve tumour. The low-grade form is commonly seen in children with 75 % of cases presenting before the age of 10 years. The less common aggressive form is seen in adults and is often fatal. About 38 % of paediatric patients with ONG have neurofibromatosis (NF)-1, and about 50 % of patients with NF1 harbour ONG. Bilateral disease is pathognomonic of NF-1 (Fig. 4). Most paediatric ONG cases involve the intraorbital and intracranial portions of the optic nerve; about 20 % may extend up to the chiasm, hypothalamus, and optic tracts. Involvement of the chiasm is common in adult ONG. Patients typically present with decreased vision and painless proptosis. Hypothalamic lesions can cause hydrocephalus. Benign ONG are slow growing tumours that do not metastasize. Imaging is mandatory to guide management and to document disease progression. The role of chemotherapy and radiotherapy (RT) in the treatment is still controversial. Long-term prognosis is excellent if the tumour is confined to the optic nerve; however, intracranial extension is associated with poorer survival rates [1, 4, 8, 25, 26].

Most ONG are low-grade pilocytic astrocytomas (WHO grade1). Histologically, these tumours are biphasic and consist of bipolar cells and multipolar cells. Bipolar cells show pilocytic processes, whereas multipolar cells may show eosinophilic granular bodies. In addition, Rosenthal fibres, microcysts, and neovascularity are commonly seen. Adult forms are usually aggressive anaplastic astrocytoma (WHO grade 3) or glioblastoma multiforme (WHO grade 4) [25, 26].

On CT and MRI, ONG show fusiform or tubular enlargement often with kinking of the optic nerve and ectasia of the optic sheath (Fig. 4). Calcification is rare. Widening of the optic canal and cystic degeneration may be seen. ONG are usually hypointense on T1W images, slightly hyperintense on T2W images and show variable enhancement (Fig. 4). MRI is the imaging modality of choice as it detects small tumours and elegantly maps out intraorbital as well as intracranial extensions. ONG can be differentiated from optic nerve sheath meningioma (ONSM) as the latter are commoner in adults, dark on T2W images, surround the optic nerve, and show avid post-contrast enhancement [1, 4, 8, 25, 26].

ONG show increased diffusion on DWI; they exhibit high ADC and low fractional anisotropy (FA) values. This is attributed to their low cellularity and low proliferative indices [11, 13, 38, 39]. Jost et al have reported on the use of DWI and dynamic contrast-enhanced imaging to calculate the diffusivity and permeability of ONG. Similar to other pilocytic astrocytomas, ONG show ADC values in the range of 1.2-2.09 × 10 ^-3^ mm^2^/s. ADC values cannot distinguish between clinically stable and clinically aggressive ONG. On the other hand, clinically aggressive ONG are seen to show significantly higher permeability values than clinically stable tumours [38]. DTI tractography can be used in the presurgical evaluation of ONG by demonstrating integrity of the optic nerve in patients with resectable lesions. Tractography can demonstrate divergence or disruption of nerve fibres. This allows for minimal post-surgical morbidity and minimal vision loss [14–16, 39]. Fillipi et al found that DTI may offer increased sensitivity over conventional imaging in evaluating the optic pathways in NF-1 patients. These patients showed statistically significant decrease in FA values and elevated mean diffusivity values as compared to age-matched controls [40]. Currently, no reliable factors have yet been identified to predict future vision loss in patients with ONG. Nevertheless, de Blank et al found that decreased FA of the optic radiations in patients with ONG was predictive of visual acuity loss during the following year and, therefore, suggested that DTI may be used as a non-invasive tool to prospectively identify those patients who will require therapy [41].

Although PET CT is not used routinely for paediatric patients, Miyamoto et al have reported very high FDG uptake in an adult patient with ONG, which turned out to be an anaplastic astrocytoma on histology. They also reported an adult patient with fibrillary astrocytoma of the optic nerve showing isointense FDG uptake. Thus, PET CT may potentially play a role in the histological grading of ONG, especially in adult patients [19].

#### Optic nerve sheath meningioma (ONSM)

ONSM is the most common primary tumour arising from the optic nerve sheath. It is a benign, slow-growing tumour, which accounts for 5 % of primary orbital tumours and 2 % of all meningiomas. It commonly occurs in females between 30-70 years of age. Hormonal factors, radiation exposure, and hereditary predisposition (abnormalities of chromosome 22) have been implicated in the aetiology. It is rare in children except in cases of neurofibromatosis (NF)-2. Primary ONSM arise from the intraorbital and intracanalicular segments of the optic nerve while secondary ONSM are intraorbital extensions of intracranial tumours. A primary ONSM may extend intracranially to involve the contralateral optic nerve. ONSM typically presents with slowly progressive painless loss of vision (with preservation of central visual field) and progressive proptosis. Although benign, these tumours have a tendency to recur. Because of their slow progression, small ONSM are often managed just by conservative follow-up imaging. Fractionated stereotactic RT is the treatment of choice for patients with preservable vision [1, 8].

ONSM arises from the arachnoid cap cells within the optic nerve sheath. Histologically, an ONSM reveals various subtypes of which the meningothelial (lobules of tumour cells), fibrous (bundles of spindle cells), and transitional types (whorls, psammoma bodies) are most common. All these are WHO grade 1 benign tumours. In benign meningiomas, tumour cells are uniform, mitoses are rare and necrosis is absent. Atypical meningiomas (WHO grade 2) have increased mitotic activity and cellularity, prominent nucleoli, and necrosis; some are of clear cell subtype. Anaplastic meningiomas (WHO grade 3) include rhabdoid/papillary subtypes and histological features of frank malignancy like extensive mitoses and necrosis [26, 42].

**Fig. 5:**
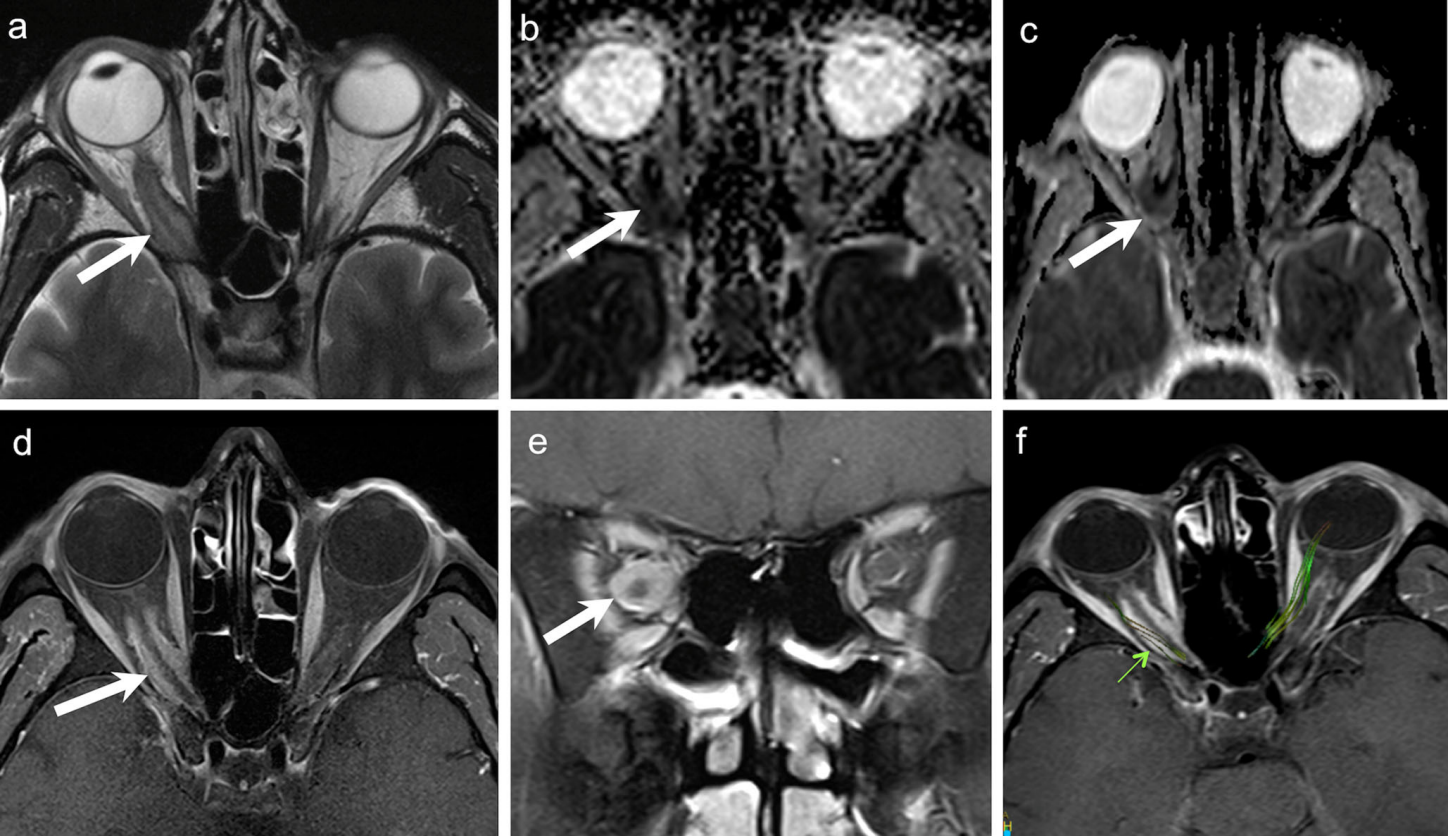
41-year-old woman with right-sided progressive vision loss and proptosis. **a.** Axial T2W image shows a moderately hpointense fusiform lesion originating from the optic nerve sheath encasing the optic nerve (arrow). **b.** Corresponding axial ADC map from RESOLVE DWI shows restricted diffusion (arrow). Note only minor image deformation. **c.** ADC map from standard EPI sequence also shows restricted lesion diffusivity (arrow); however, note major image deformation due to susceptibility artefacts. **d.** Axial contrast enhanced T1W image reveals strong fusiform enhancement along the optic nerve (“tram track sign”, arrow). There was no extension through the orbital apex intracranially. **e.** Coronal fat saturated T1W image displaying concentric enhancement of the tumour around the compressed optic nerve creating a characteristic “bull’s eye” appearance (arrow). Imaging findings are in keeping with an ONSM. **f.** DTI 3D tractography reconstruction of the optic nerves reveals normal fibres on the left (fibers are depicted in green due to their anterior- posterior course) and major fibre atrophy on the right (arrow)

The diagnosis of ONSM depends on cross-sectional imaging. NECT commonly shows tubular thickening and calcification of the optic nerve sheath complex. An enlarged optic nerve canal and hyperostosis may be seen. Although MRI is less sensitive than CT for detection of calcifications, it is ideal for assessing the intracanalicular and intracranial extension of ONSM. ONSM demonstrates similar intensity as the optic nerve on T1W and T2W images. Fat-saturated, thin-section CEMRI images show a tubular enhancing mass around the isointense optic nerve (tram track sign on axial images/target sign on coronal images) (Fig. 5). ONG is the closest imaging differential; however, ONG is more common in children, does not calcify, expands the optic nerve, often associated with other stigmata of NF-1, and may extend intracranially along the optic pathways [1, 8]. Abnormal enhancement of the optic nerve sheath causing the tram track sign or the target sign appearance on cross-sectional imaging may also be seen in the setting of lymphoma, leukemia, and inflammatory pseudotumour or can be caused by tumour seeding into the subarachnoid space (as the optic nerve sheath communicates with the intracranial subarachnoid space).

Because of its hypercellular nature, ONSM often shows restricted diffusion with low ADC values on DWI [9–12] (Fig. 5). Sepahdari et al have reported ADC values in the range of 0.79-1.04 × 10^−3^ mm^2^/s for ONSM [9]. Zang et al have illustrated a case of tractography demonstrating optic nerve displacement and atrophy due to an invasive orbital meningioma [15].

It has been reported that WHO grades 2 and 3 dural meningiomas show higher FDG uptake than grade 1 tumours. Thus, FDG uptake correlates with the proliferative activity of meningiomas [43, 44].

### Conal-intraconal compartment

#### Cavernous hemangioma

Cavernous hemangioma is the most common benign orbital tumour-like condition in adults. According to the classification of vascular malformations by the International Society for the Study of Vascular Anomalies (ISSVA), cavernous hemangioma is a venous or low flow malformation [1–4, 8]. It is more common in women and usually seen in the 2nd–4th decades. It is typically intraconal and presents with slowly progressive, unilateral proptosis, and/or diplopia. Occasionally, compression of the optic nerve may result in mild visual deficit. Unlike childhood capillary hemagiomas, cavernous hemangiomas do not show cellular proliferation nor do they have a prominent arterial supply. In fact, they are isolated from the orbital vascular system. Asymptomatic tumours are usually observed conservatively. Surgical resection is performed to correct visual disturbance and cosmetic problems. Visual prognosis is excellent in most cases [1, 2, 45, 46].

Histologically, cavernous hemangiomas consist of large blood-filled spaces lined by flattened endothelial cells and separated by scant fibrous tissue. Stagnant circulation may result in venous thrombosis. Dystrophic calcification is common [26, 45]. Cavernous hemangiomas can coexist with other vascular malformations of the orbit like venous varix and lymphangioma [47].

**Fig. 6:**
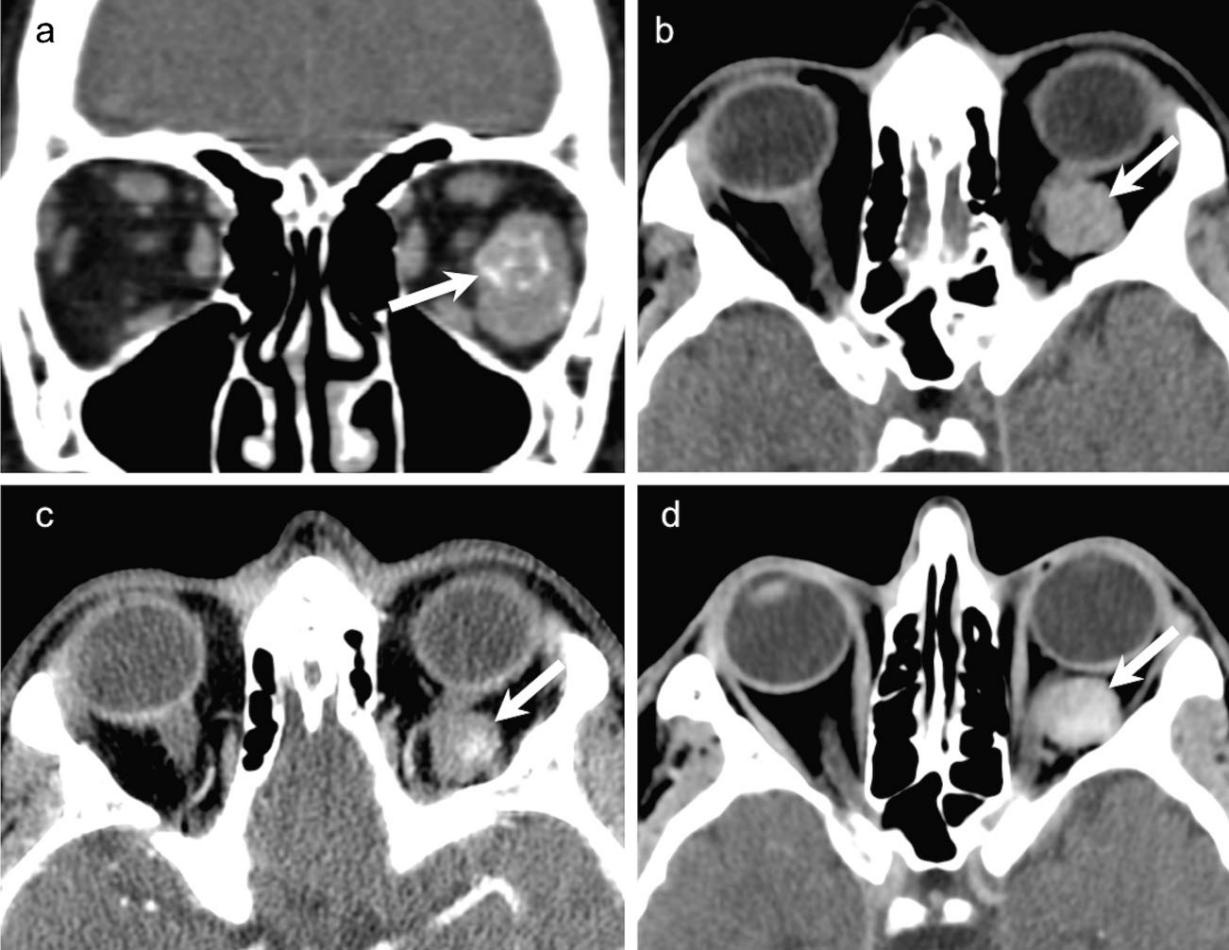
Orbital hemangioma as seen on CT in two different patients. **a.** Coronal NECT in a 30-year-old male patient shows a well-circumscribed intraconal mass (arrow) with calcified phleboliths. **b-d.** 50-year-old male patient with an incidentally diagnosed left orbital cavernous hemangioma on an angio-CT performed for stroke. **b.** NCECT shows a nonspecific intraconal lesion (arrow) without phleboliths. Arterial phase (**c**) demonstrates initial patchy enhancement (arrow) followed by progressive filling of the lesion in the venous phase (**d**)

**Fig. 7:**
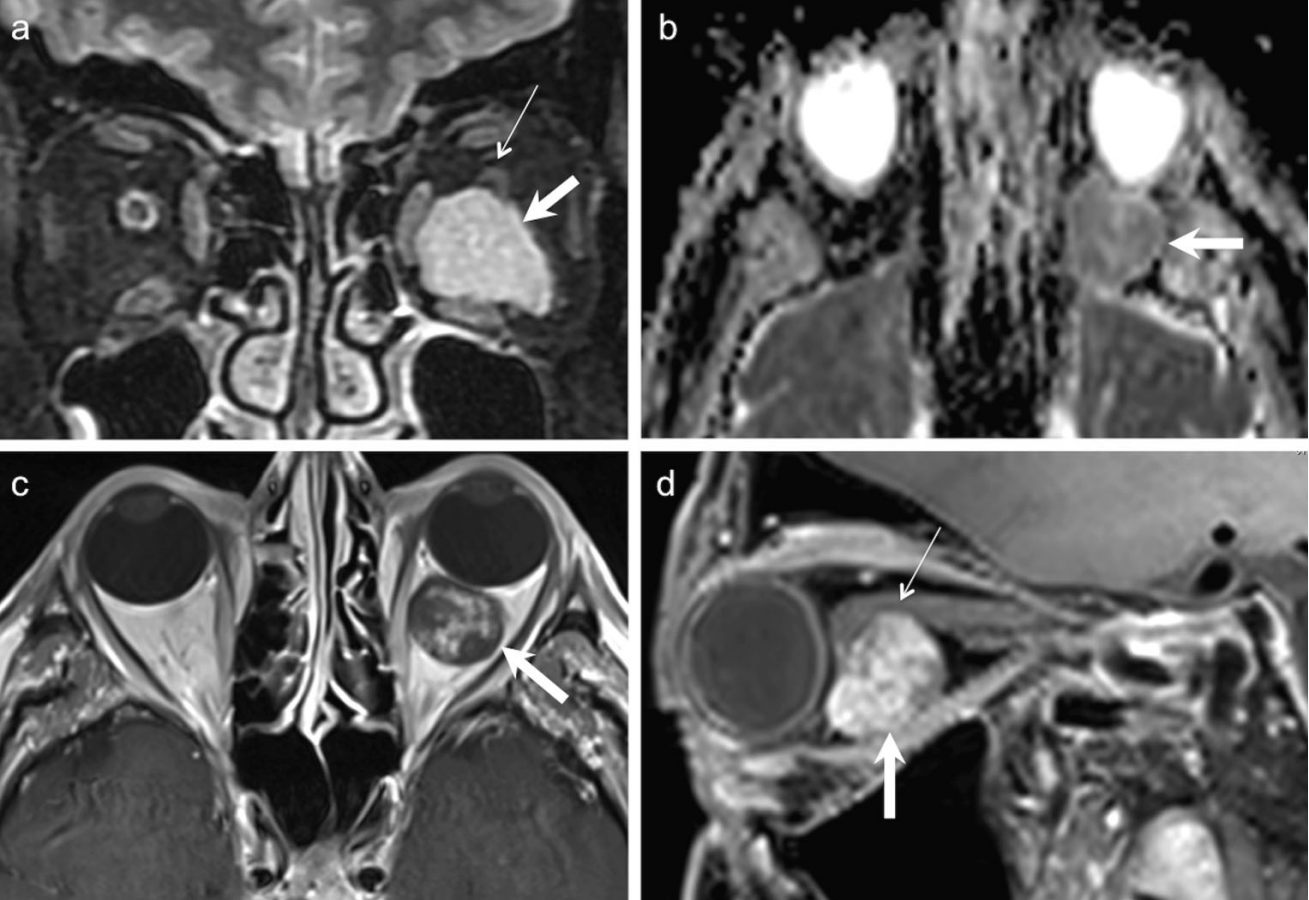
60-year-old man with left orbital cavernous hemangioma. Coronal STIR image (**a**) shows a well-circumscribed hyperintense intraconal mass (arrow) causing superior displacement of the left optic nerve (thin arrow). **b.** ADC map shows moderately hypointense signal within the mass with an ADC value of 1.4 × 10 ^−3^ mm^2^/s. **c.** Axial contrast-enhanced TIW image shows initial patchy enhancement. **d.** Sagittal reformatted image from a contrast-enhanced 3D VIBE acquisition obtained after the T1W sequence shows characteristic progressive filling (arrow). Major mass effect on the optic nerve (thin arrow). The patient underwent surgery, which confirmed the diagnosis of cavernous hemangioma

On NECT, a cavernous hemangioma is seen as a well-circumscribed, dense intraconal mass. It is usually seen separately from the optic nerve and extraocular muscles CECT may show patchy or uniform enhancement (Fig. 6). Phleboliths, if present, help to confirm the diagnosis (Fig. 6). On MRI, the lesion typically appears iso- to hypointense on T1W images and hyperintense on T2W images, although some hemangiomas may display moderately high/low signal intensity. Some scattered hyperintense areas on T1W images may indicate thrombosis. Fat-suppressed, dynamic CEMRI shows initial patchy enhancement followed by homogeneous enhancement in the delayed phase (Fig. 7). Imaging differentials include venous varix and, less often, schwannoma. A venous varix shows intermittent intralesional flow and exophthalmos on Valsalva manoeuvre [1, 2, 45, 46]. Schwannomas also appear well-circumscribed, iso-hypointense on T1W images, and hyperintense on T2W images. However, the spread pattern on dynamic CEMRI can help to distinguish between schwannoma and cavernous hemangioma. In the early phase, enhancement in hemangiomas starts at one point, whereas it starts from a wide area in schwannomas [48, 49].

Cavernous hemangiomas appear bright on DW images with mean ADC values ranging from 1.23 × 10^−3^ mm^2^/s to 1.39 × 10^−3^ mm^2^/s [9, 10, 12] (Fig. 7). Razek et al have reported that schwannomas have mean ADC value of 1.92 × 10^−3^ mm^2^/s, which is statistically different from that of cavernous hemangiomas [12].

Scinitigraphy using technetium 99 m-labelled red blood cells can help in the differential diagnosis of cavernous hemangioma from other imaging mimics [50]. Miyamoto et al have described two cases of adult orbital cavernous hemangiomas showing isointense FDG uptake on PET/CT [19].

### Extraconal compartment

#### Dermoid

Dermoids are the most common congenital orbital lesions, accounting for about 25 % of all orbital biopsies. They arise from epithelial sequestration, usually at the zygomaticofrontal and frontoethmoidal sutures. They are typically seen in the extraconal region, superolaterally, between the globe and the orbital periosteum. They are slow growing lesions; however, they may occasionally rupture and mimic acute inflammation. Small dermoids do not require immediate treatment and are usually removed within the 2nd or 3rd year of life. Complete excision without rupturing is necessary in order to avoid inflammatory reactions or local recurrences [1, 26, 51].

Macroscopically, orbital dermoids are round or oval-shaped, encapsulated tumours, containing various skin appendages and fatty material. Histologically, a dermoid is lined by squamous epithelium and may contain hair follicles, sebaceous glands as well as keratinous debris [26, 51].

**Fig. 8:**
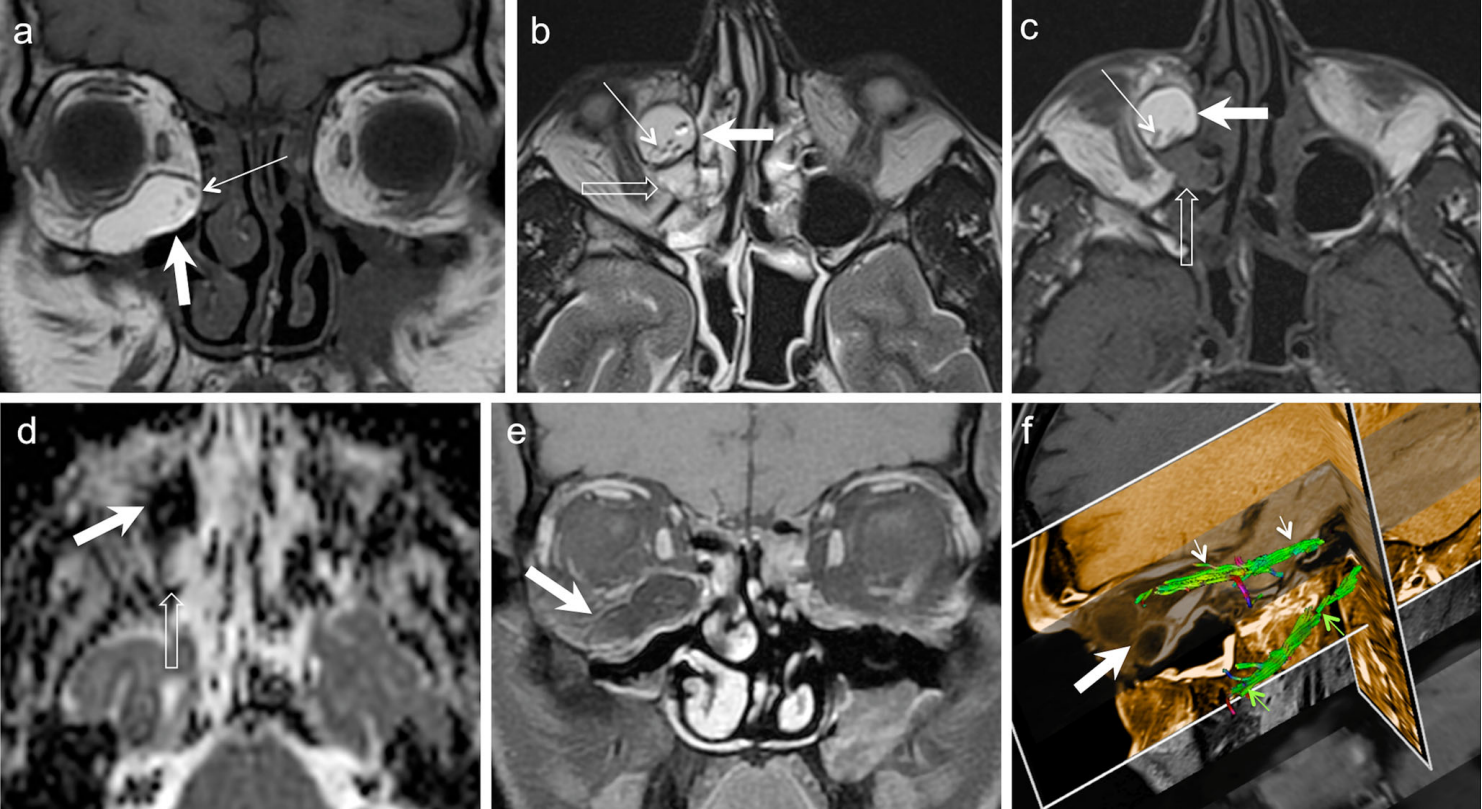
42-year-old male with a histologically proven right orbital dermoid. Coronal T1W (**a**), axial T2W (**b**), axial T1W (**c**), axial ADC map (**d**), and coronal FS contrast-enhanced T1W (**e**) images show an orbital lesion with an anterior component containing fatty tissue (thick white arrows) and a posterior component containing non-fatty elements (hollow arrows). There are some fluid droplets in the anterior component (thin arrows). Note minor capsular enhancement after gadolinium administration. The ADC values are very low in the anterior part of the lesion (ADC = 0.1 × 10 ^−3^ mm^2^/s) due to fat and they are very high in the posterior component (ADC = 1.8 × 10 ^−3^ mm^2^/s) due to fluid. **f.** DTI 3D tractography reconstruction of the optic nerves (green) viewed from above and from the left. Right optic nerve fibres (white thin arrows) and left optic nerve fibres (green thin arrows) are normal and have similar FA and ADC values. Thick arrow points to the dermoid

In addition to clinical examination, which is sufficient for most superficial dermoids, US scans help to demonstrate a sharply outlined lesion with a capsule and low-reflectivity contents. CT or MRI are rarely necessary. NECT shows a well circumscribed, cystic tumour of low or fat density. Fat-fluid levels and calcifications may be seen. Bony scalloping of the lacrimal fossa may occur due to pressure effect. On MRI, dermoids typically appear hyperintense on T1W and T2W and hypointense on STIR (Fig. 8). Rim enhancement is rare [1, 26, 51]. Diffusion in dermoids may be variable depending upon their contents. Lope et al and Sepahdari et al have described restricted diffusion in orbital dermoids [9, 13], whereas Razek et al have described ADC values in the range of 1.68-1.95 × 10^−3^ mm^2^/s for head-neck dermoids [52]. Most often, the detection of macroscopic fat in dermoids is diagnostic and DWI adds no additional value (Fig. 8).

Cephalocoeles can occur in the fronto-orbital region near the midline and can mimic dermoids. Cephalocoeles show a track leading to the anterior cranial fossa, contain cerebrospinal fluid and show no fat content [51].

### Lacrimal gland tumours

Lacrimal gland tumours are classified as epithelial and non-epithelial lesions. Lacrimal gland epithelial tumours are similar to salivary gland tumours and constitute 40–50 % of all lacrimal masses. Half of these are benign mixed tumours (BMT), while the other half are malignant masses. Adenoid cystic carcinoma (ACC) is the most common lacrimal gland malignancy, followed by carcinoma ex-pleomorphic adenoma, adenocarcinoma, and mucoepidermoid carcinoma. Lymphoma, inflammatory conditions, and non-carcinomatous metastases constitute the non-epithelial lesions.

#### Benign mixed tumour (BMT)

BMT or pleomorphic adenoma originates mainly from the orbital lobe of the lacrimal gland. It is seen in middle-aged patients (40-50 years) without gender predilection. Clinical signs include a painless, slow-growing mass in the lateral orbit, usually persisting for more than 12 months. Proptosis may be seen. Although benign, these tumours can show recurrence and malignant transformation (carcinoma ex-pleomorphic adenoma), primarily in cases where only a biopsy or incomplete excision were performed. Hence, meticulous surgical excision and careful pathological examination for capsular invasion is mandatory [53, 54].

**Fig. 9:**
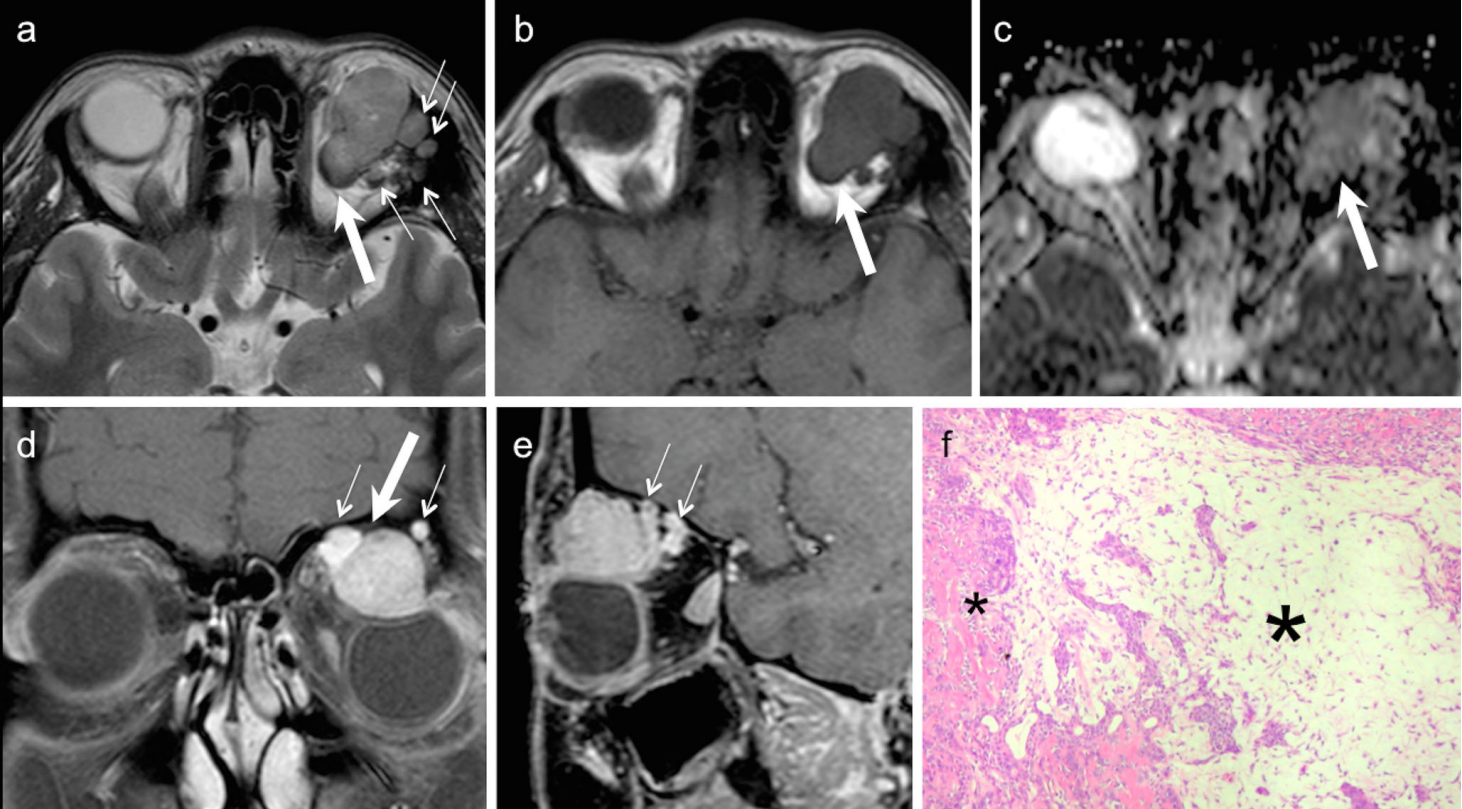
42-year-old female patient with BMT of the left lacrimal gland. **a.** Axial T2W MR image shows a well-circumscribed polypoid mass (thick arrow) of moderately hypointense signal involving the left lacrimal gland. Note small satellite nodules (thin arrows). **b**. Corresponding T1W image shows non-specific lesion hypointensity (arrow). **c.** ADC map reveals increased diffusion (ADC = 1.6 × 10 ^−3^ mm^2^/s). **d.** Coronal contrast-enhanced FS T1W MR image of the same patient demonstrates strong enhancement within the mass (thick arrow) and within the peripheral nodules (thin arrows). **e.** Sagittal reformatted 0.6 mm thin image from contrast-enhanced 3D VIBE better illustrates scalloping of the orbital roof by the peripheral “grape-like” nodules (thin arrows). **e.** Photomicrograph of the surgical specimen (original magnification 100x, H&E stain) illustrates the characteristic histological features of pleomorphic adenoma with medium sized cells with an eosinophilic cytoplasm and myoepithelial cells (small asterisk) partly surrounded by a myxoid matrix (large asterisk). The peripheral nodules seen on MRI corresponded histologically to pseudopodia and satellite nodules. Bony invasion by pseudopodia was confirmed histologically

BMT arise from the ductal system of the gland. These encapsulated tumours contain interspersed epithelial and stromal components. Necrosis, hyalinization, myxoid, and mucinous degeneration may be seen [26, 53, 54]. Pseudopodia and satellite tumours have been described histologically in more than 50 % of BMT arising in the parotid gland, these histological features being associated with a higher risk of recurrence [55]. Pseudopodia, satellite lesions, and bony remodelling can equally be seen in BMTs arising in the lacrimal glands; they should not be misinterpreted as a feature of malignancy (Fig. 9). On CT, BMT are seen as well-circumscribed, round-oval masses with varying attenuation depending upon their composition and cellularity. Highly cellular masses appear homogeneous. Cystic degeneration may give rise to hypodense/inhomogeneous appearance. Lacrimal fossa deformation and calcifications may be seen on bone-window CT. On MRI, a heterogeneous signal is identified, especially on T2W images with moderate/heterogeneous or homogenous contrast enhancement (Fig. 9). Infiltration of the adjacent orbital tissue and poorly defined margins suggest malignant transformation [53, 54]. Motoori et al proposed that the detection of myxomatous tissue on T2W, inversion recovery, DWI, and dynamic contrast-enhanced sequences help to differentiate BMT from other malignant tumours [56].

Similar to salivary gland BMT, lacrimal gland BMT do not show restricted diffusion (Fig. 9) [56–58]. Sepahdari et al have reported a single BMT of the lacrimal gland with ADC value of 1.37 × 10 ^−3^ mm ^2^/s [9], Razek et al have reported ADC ranges in BMT between 1.62-1.76 × 10 ^−3^ mm^2^/s [12], whereas Elkhamary et al have reported ADC ranges between 1.18-1.23 × 10 ^−3^ mm^2^ /s [58]. These ADC values are significantly higher than the malignancy threshold value of 1.0 × 10^−3^ mm^2^/s [9, 10, 12, 56–58]. Thus, ADC values appear to be highly sensitive and specific in differentiating BMT from malignant tumours of the lacrimal gland.

#### Malignant epithelial lacrimal gland tumours

**Fig. 10:**
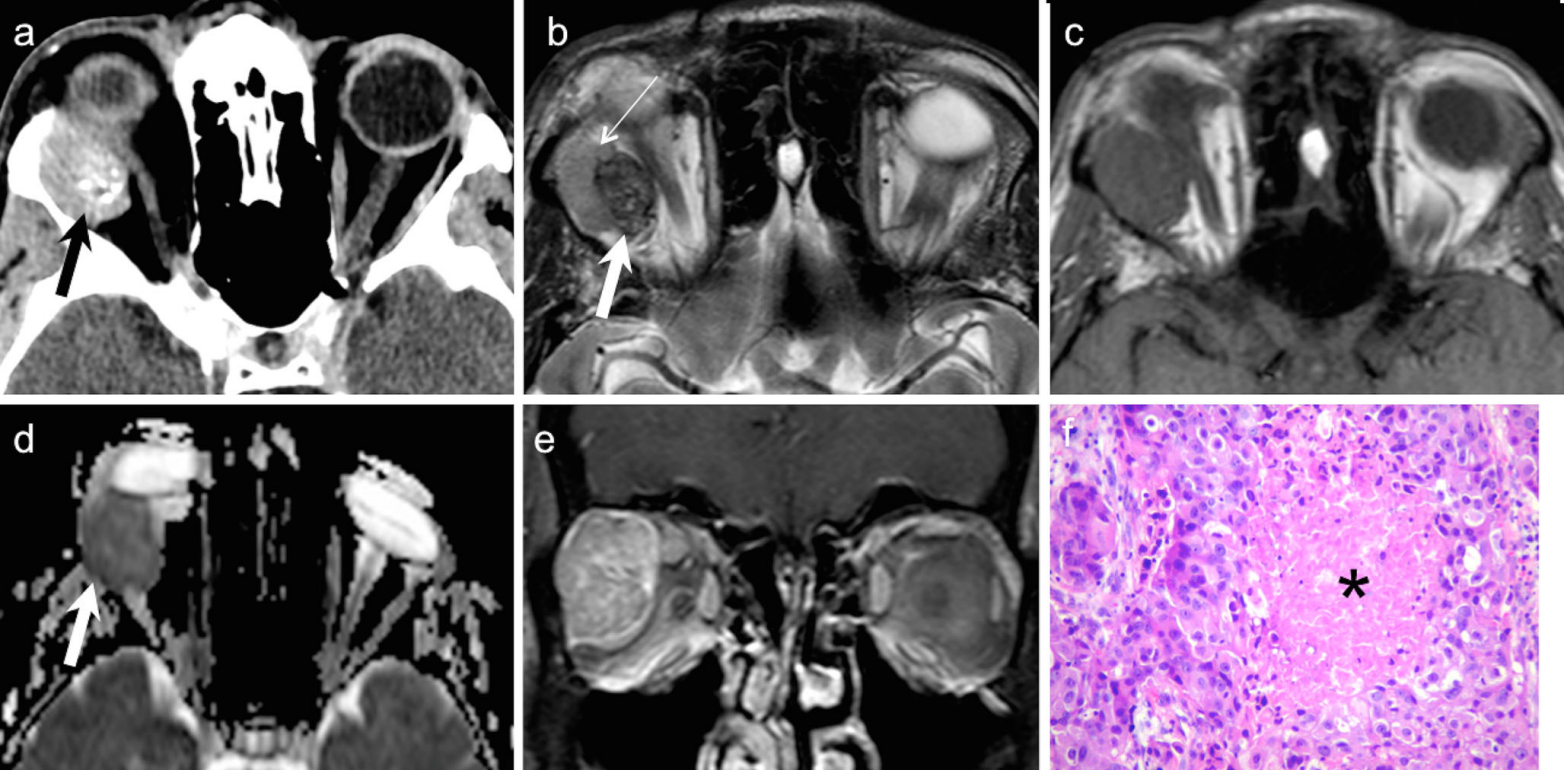
65-year-old male patient with undifferentiated ductal carcinoma of the right lacrimal gland (mammary analog secretory type). **a.** Axial NECT of the orbits shows a well-circumscribed, hyperdense mass with stippled calcifications involving the right lacrimal gland. The calcifications were misdiagnosed as phleboliths, which led to the initial diagnosis of a cavernous hemangioma. **b.** Axial T2W MR image of the same patient shows that the lacrimal gland mass has a very hypointense posterior component (thick arrow) and an anterior moderately hypointense portion (thin arrow). **c.** Corresponding axial T1W MR image shows that the mass is isointense to the rectus muscles. **d.** ADC map reveals restricted diffusion (ADC = 0.9 × 10 ^−3^ mm^2^/s), suggesting a malignant tumour. **e.** Coronal FS contrast-enhanced T1 W image shows moderate tumour enhancement and lobular appearance. **f.** Photomicrograph (original magnification 100x, H&E stain) shows a highly cellular tumour with atypical nuclei and mitoses and areas of necrosis (asterisk). There were numerous areas of microscopic perineural spread and lymphatic invasion not detected by imaging

Approximately 50 % of epithelial lacrimal gland tumours are malignant. Malignant tumours of epithelial origin include adenoid cystic carcinoma (ACC), mucoepidermoid carcinoma, adenocarcinoma, squamous cell carcinoma, and undifferentiated carcinoma types, such as the mammary analog secretory carcinoma of salivary origin (Fig. 10). Overall, these tumours are quite rare, accounting for less than 5 % of all lacrimal gland lesions. Nevertheless, malignant lacrimal gland tumours need to be recognized at an early stage, as they tend to have a high morbidity and mortality. ACC is a high-grade malignancy accounting for 29 % of all epithelial lacrimal gland tumours and as many as 50 % of all malignant epithelial lacrimal gland neoplasms. Its incidence peaks in the 4th decade. Patients present with a hard mass in the upper lateral orbit, often with pain caused by perineural spread or bony invasion. Perineural spread indicates poorer prognosis. Radical resection with wide margins is performed for small, low-grade tumours. Survival rates vary from 5 years in 40 % of patients to 15 years in 58 % of patients [1, 2, 53, 54]. Histologically, these tumours are characterized by absence of a mesenchymal matrix. They show different histological types like tubular, comedo-carcinoma, basaloid, or cribriform patterns. The cribriform pattern consists of sheets of basaloid epithelial cells and surrounding spaces of varying shapes giving rise to a characteristic Swiss cheese appearance [26, 53, 54].

**Fig. 11:**
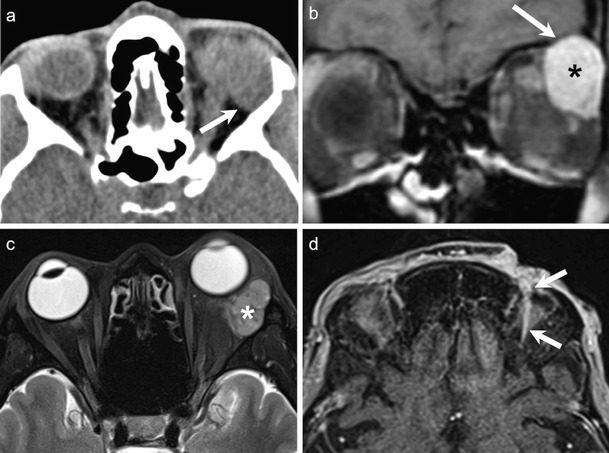
50-year-old male patient with histologically proven ACC of the left lacrimal gland. Axial NECT of the orbits shows a well-circumscribed, slightly hyperdense mass (arrow) involving the left lacrimal gland. There is suggestion of minimal adjacent bony scalloping. **b.** Coronal contrast-enhanced FS TIW image of the same patient shows avid, homogeneous, contrast enhancement within the mass (asterisk), mimicking a cavernous hemangioma. There is thinning of the overlying left frontal bone (arrow). **c.** Axial T2W image of the same patient showing intermediate signal within the mass (asterisk) suggesting high cellularity, a feature that is uncommon in hemangioma. The patient underwent surgery, which revealed ACC with multiple areas of microscopic perineural spread not detected by imaging. **d.** Contrast-enhanced axial T1W image obtained in a different patient 10 years after surgery of an ACC of the left lacrimal gland shows macroscopic perineural tumour recurrence along the supraorbital nerve (arrows). The findings were confirmed histologically

CT shows non-specific findings; often a well or poorly circumscribed mass involving the lacrimal gland with associated bony destruction in 70 % cases (Fig. 11). Low-grade tumours may exactly mimic BMT with a well-circumscribed margin and no bony destruction. Intratumoural calcifications are more common in ACC, in adenocarcinoma and in undifferentiated epithelial tumours (Fig. 10) than in a BMT and should not be misinterpreted as phleboliths. ACC often appears hypointense on T1W images, hypo- or hyperintense on T2W images and shows prominent post-contrast enhancement (Fig. 11). Fat-saturated CEMRI is ideal for local tumour staging and for evaluating perineural spread [1, 2, 53, 54]. In general, most ACC will show perineural spread at microscopic examination; however, radiologically, perineural spread may remain undetected unless involvement of major nerve trunks, such as where the supraorbital nerve occurs (Fig. 11).

Elkhamary SM et al have reported 0.8 × 10 ^−3^ mm^2^ /s as the mean ADC value for ACC (which is significantly lower than BMT and other benign lesions), but significantly higher than lymphomas (0.6 × 10 ^−3^ mm^2^/s) [58]. ADC values can help to differentiate between carcinomas, lymphomas, metastases, and inflammatory disorders (described in subsequent sections) [1, 9–12, 53, 54, 58]. Nevertheless, ACC is often indistinguishable from other malignant epithelial tumours of the lacrimal gland on MRI with DWI (Fig. 10) unless perineural spread, which is more common in ACC, is detected (Fig. 11). Occasionally, mucoepidermoid carcinomas may show high T1 signal due to their mucin content.

ACC is commonly hypermetabolic on PET CT. Baek et al have reported that PET CT was successful in detecting both the primary lesion and distant metastases of lacrimal gland ACC [17–19, 59].

#### Rhabdomyosarcoma (RMS)

RMS is the most common malignant mesenchymal tumour of childhood. The orbit is the most common location in the head-neck with 40 % of tumours appearing there. The incidence peaks between 5-10 years of age, as shown by positron emission tomography, with a slight predilection for boys. RMS typically arises in the extraconal compartment; however, intraconal extension is known. It presents with rapidly progressive proptosis, ptosis, or signs of inflammation often prompting urgent imaging. It is an aggressive tumour and commonly infiltrates into the adjacent sinuses, orbital fissures, cavernous sinus, and middle cranial fossa. Hematogenous spread can occur to the lungs. Surgery and chemotherapy used in combination may achieve good survival rates. RT is avoided because of the potential risk of cataract, encephalopathy, and radiation-induced sarcomas [1, 2, 4, 26, 60].

RMS is believed to arise from primitive mesenchymal cells. RMS can be divided histopathologically into embryonal, pleomorphic, alveolar, and botryoid subtypes. The alveolar subtype is the most anaplastic variant characterized by tumour cells spreading along soft-tissue septa. The rhabdomyoblast is the diagnostic cell in all types which shows granular eosinophilic cytoplasm with thick and thin filaments. Conventional H&E staining is complimented with immunohistochemistry to detect myoglobin and desmin which support the diagnosis [26, 60].

**Fig. 12:**
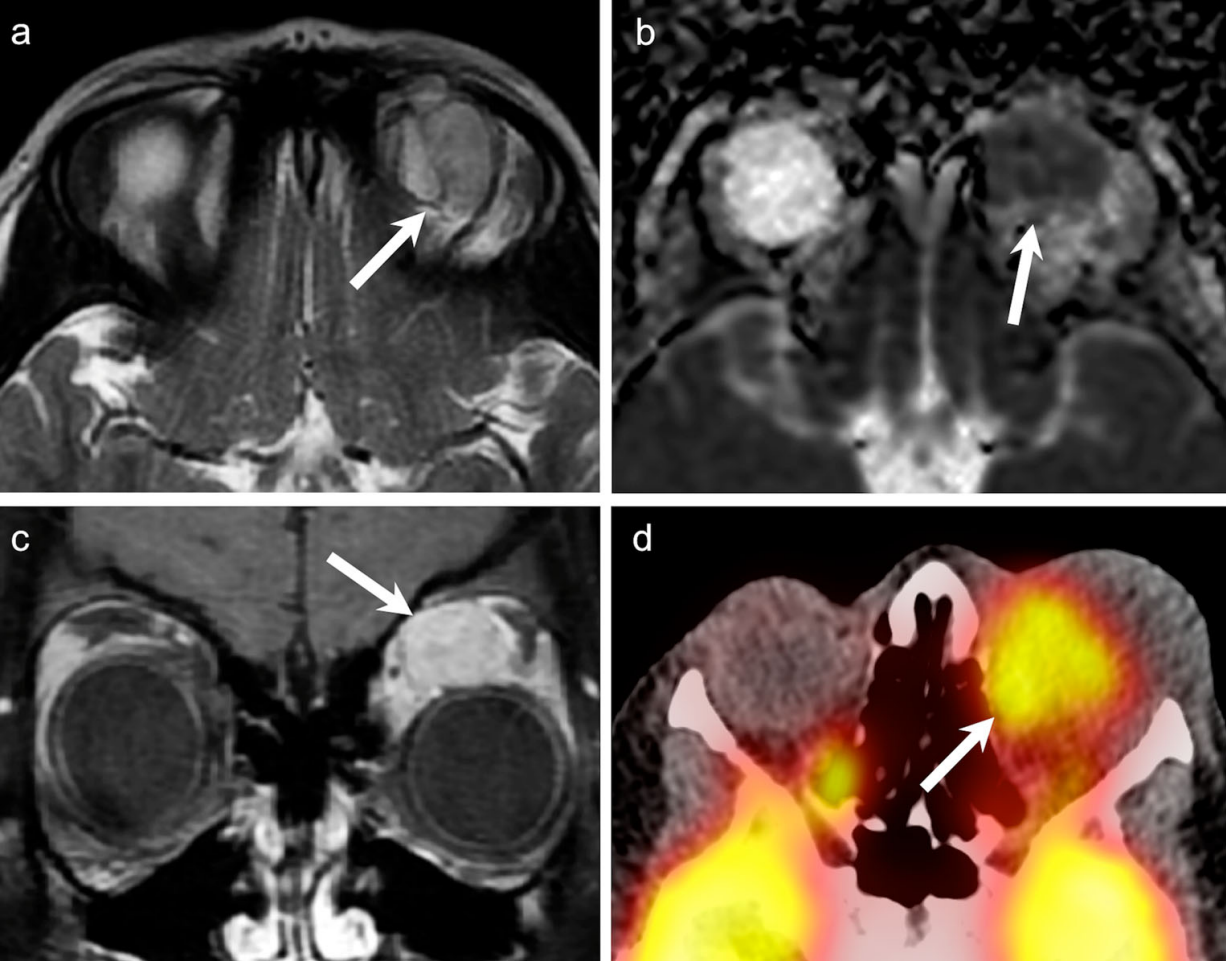
4-year-old girl with a left orbital RMS. **a.** Axial T2W MR image of the orbits shows a conal-extraconal polypoid mass of moderately low signal intensity located superior to the left globe. **b.** ADC map shows restricted diffusion (ADC = 1 × 10 ^−3^ mm^2^/s), suggestive of malignancy. **c**. Coronal contrast-enhanced FS T1W MR image shows avid enhancement within the lesion. **d**. Axial FDG PET/CT image of the same patient shows high SUVs (SUVmean = 6, SUVmax = 9). Although the lesion may mimic a cavernous hemangioma on the T1W and T2W images, the low ADC and the high FDG uptake strongly suggest a malignant tumour. Histology revealed embryonal rhabdomyosarcoma

CT and MR are often used in combination to assess tumour size, extraorbital extension, bony destruction and intracranial involvement. RMS appears isodense to muscle on NECT and usually shows significant enhancement on CECT. It appears iso-intense to muscle on T1W images, hypo or hyperintense on T2W images and shows marked enhancement on CEMRI (Fig. 12). Necrosis and calcification is uncommon [1, 2, 4, 26, 60].

Many benign and malignant entities may mimic the imaging features of RMS; however, the presence of unilateral, rapidly progressive proptosis in a child must always raise concern for RMS. Orbital cellulitis may show similar clinical features. On imaging, both conditions may show an orbital mass with adjacent paranasal sinus involvement. Fever, leukocytosis, and orbital fat stranding/abscess formation suggest infection. Although capillary hemangiomas manifest in a younger age-group (12-18 months), they may mimic a very vascular RMS. Capillary hemangiomas often show multiple flow-voids, avid contrast enhancement, and high flow on time-resolved MR angiography. Associated cutaneous hemangiomas are seen in one third of cases. In cases with equivocal clinical and imaging findings, a biopsy is necessary to reach the diagnosis. Langerhans cell histiocytosis may present with orbital involvement in 23 % cases. It can appear as an aggressive soft tissue mass with bony invasion, similar to RMS. It may be associated with diabetes insipidus. Again, a biopsy may be necessary to differentiate between the two. Neuroblastoma metastases to the orbit can mimic RMS on imaging. The finding of a primary tumour in the retroperitoneum or posterior mediastinum helps to arrive at the correct diagnosis [60]. Lymphoma is rare in children accounting for less than 5-10 % of orbital lesions. Unlike RMS, it commonly involves the lacrimal gland, appears hypointense on T2W images, encases rather than distorts the globe and shows even lower ADC values than RMS [4, 13, 60] .

Sepahdari et al have reported 0.72 × 10 ^−3^ mm^2^/s as the mean ADC value in a cohort of 12 cases of orbital RMS [10] (Fig. 12). Lope et al have stated that the low ADC values of RMS can help to differentiate them from haemangiomas which usually show high ADC values [13]. Hassold et al have reported a case of orbital RMS where low ADC value within the recurrent tumour (0.62 × 10 ^−3^ mm^2^/s) helped to differentiate it from post-therapeutic changes [61].

PET CT is a valuable adjunct for the grading and staging of paediatric sarcomas [62]. FDG uptake is known to correlate with the tumour grade of mesenchymal sarcomas [62, 63] (Fig. 12). PET CT has been deemed superior at detecting osseous and nodal metastases of RMS than conventional imaging [62, 64]. Whole-body MRI has shown comparable results as PET CT, and more accuracy as compared to skeletal scintigraphy in the detection of osseous metastases of RMS [65]. MRI PET may potentially improve the staging, follow-up and post-treatment assessment of RMS [22].

#### Lymphoma

Lymphoma of the ocular adnexa is the most common orbital malignancy accounting for 55 % of adult malignant orbital tumours. This is a heterogeneous group of tumours composing approximately 1-2 % of non-Hodgkin’s lymphomas (NHL) and 8 % of extranodal mucosa-associated lymphoid tissue (MALT) lymphomas. Highest incidence is seen in the 6th–7th decade. Lymphoma can be primary to the orbit or secondary to systemic disease. Secondary ocular involvement occurs in 2-5 % of patients with advanced systemic NHL. About 75 % of patients with primary orbital lymphoma eventually develop systemic disease. Patients with Sjögren’s syndrome have a higher risk of developing extranodal NHL as compared to age-, race-, and sex-matched controls. Sjögren’s syndrome, either primary or secondary (due to rheumatoid arthritis, systemic lupus erythematosus, or progressive systemic sclerosis), is an autoimmune disease of the exocrine glands characterized by lymphocytic infiltration of the affected glands. About 6 % of patients with Sjögren’s syndrome show clinically evident NHL manifestations. Most NHL seen in patients with Sjögren’s syndrome are of B cell lineage and can occur in the salivary glands, lacrimal glands, lymph nodes, lung, and thyroid. In the orbit, NHL can be unilateral or bilateral. In the orbit, the anterior extraconal space and lacrimal gland are most commonly involved. Involvement of the lacrimal gland is seen in about 7 % of all NHL cases associated with Sjögren’s syndrome and manifests clinically with lacrimal gland enlargement. Other clinical manifestations of orbital lymphoma include painless proptosis and motility disturbances. Whole-body staging is necessary when orbital lymphoma is diagnosed and is most often done with FDG PET CT. Low-grade tumours respond well to RT while chemotherapy is necessary for high-grade or systemic disease [1, 4, 18, 66, 67].

Histologically, ocular adnexal MALT lymphomas are characterized by a heterogeneous cell population, consisting of monocytoid, plasmacytoid, and centrocyte-like cells, with occasional blasts in the marginal zone surrounding lymphoid follicles. Pathognomonic histological features include “follicular colonization” and the formation of “lymphoepithelial lesions” through the invasion of neighbouring epithelial structures by nests of MALT lymphoma cells. Immunophenotypically, orbital MALT lymphomas show dense CD 20^+^, CD 10^−^and CD 23^−^ B-cell lymphocytic infiltrates which helps to differentiate them from benign lymphoproliferative disorders and other small B-cell lymphomas. MALT lymphomas are typically negative for CD5, which helps to differentiate them from mantle-cell lymphomas [26, 67].

**Fig. 13:**
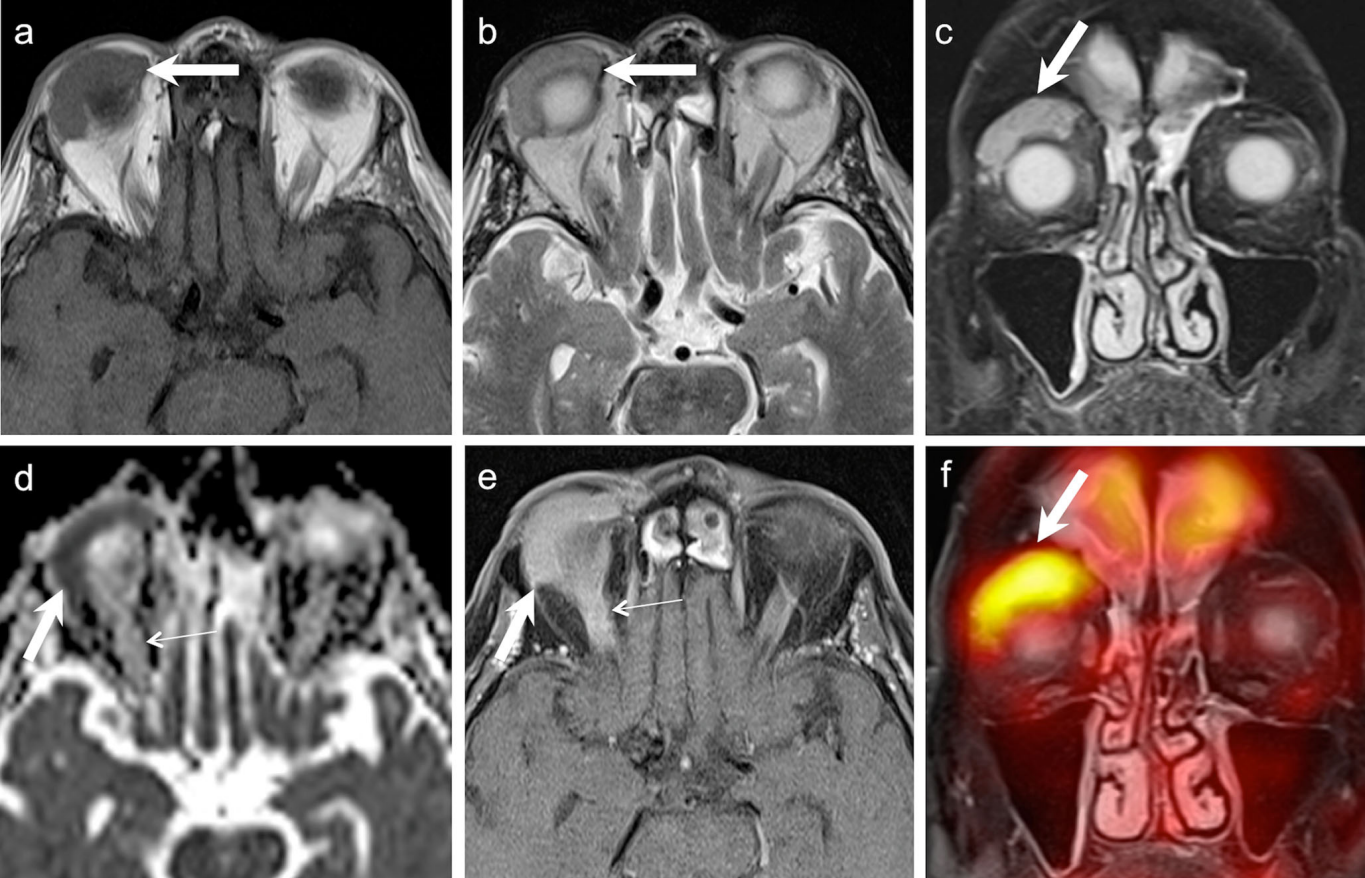
80-year-old male patient with lymphoma of the right orbit. **a.** Axial T1W image of the orbits shows a hypointense, well-demarcated anterior conal-extraconal lesion (arrow). Axial T2W (**b**) and coronal STIR (**c**) images demonstrate intermediate signal intensity of the bulky mass (arrows). Note homogenous aspect. ADC map (**d**) reveals restricted diffusion (arrow) with very low ADC values (ADC = 0.6 × 10 ^−3^ mm^2^/s) characteristic of lymphoma. Axial contrast enhanced T1W image (e) shows homogenous enhancement of the mass (arrow). There is enhancement of the superior rectus muscle (thin arrow) suggesting possible involvement. However, the ADC map clearly shows that the superior rectus muscle has no restricted diffusion (thin arrows in d and e). **f.** Coronal fused PET and STIR image from FDG PET/MRI reveals high tracer uptake within the mass (SUVmean = 4.8, SUVmax = 6.7). No other lymphoma manifestations were detected in the body

**Fig. 14:**
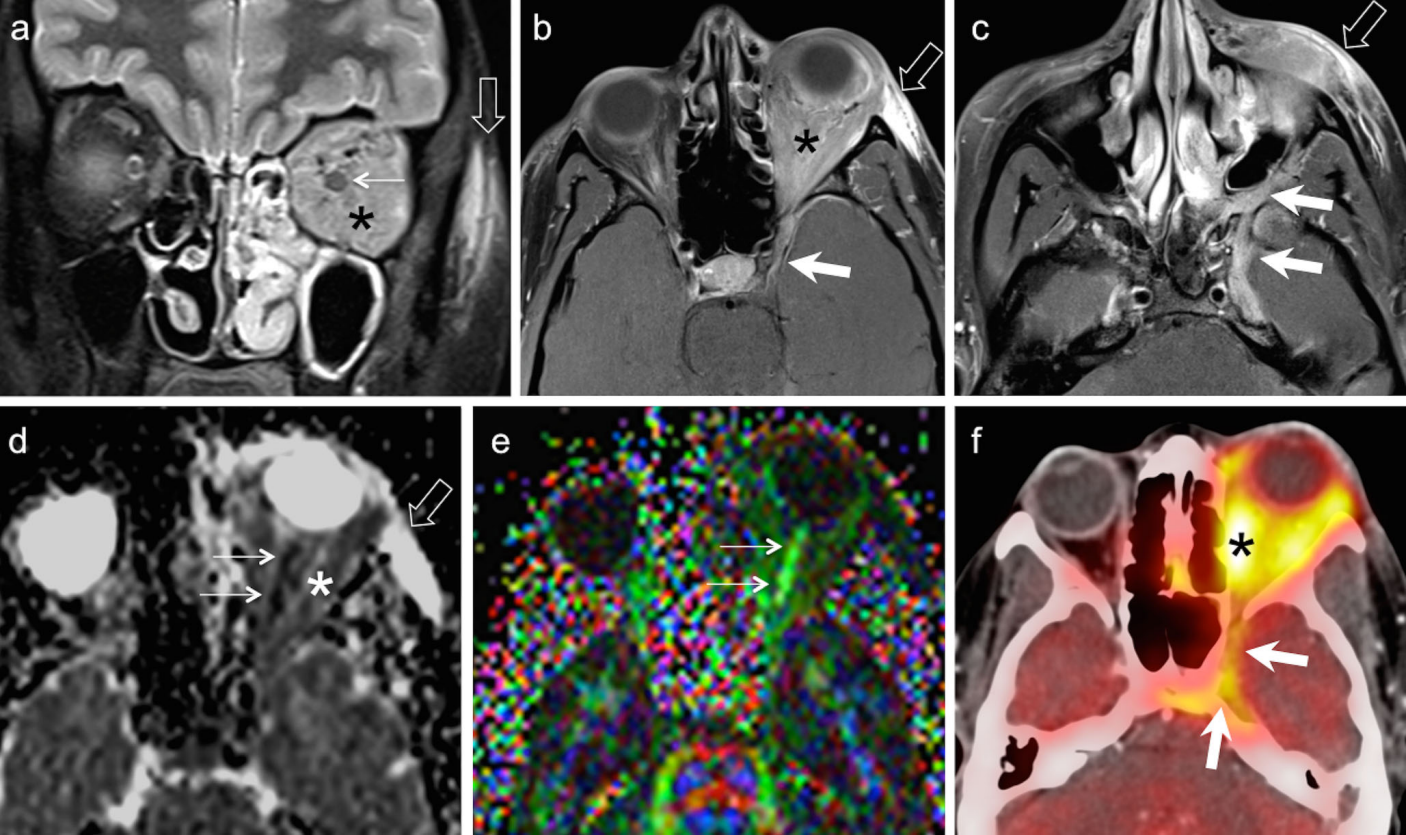
35-year-old female patient with painful proptosis, loss of vision, and subcutaneous facial swelling. Biopsy of the face performed in an outside institution suggested inflammatory pseudotumour. **a.** Coronal STIR image shows an ill-defined, moderately hyperintense lesion involving the entire left orbit (asterisk) and encasing the optic nerve (thin arrow). The subcutaneous hyperintense, poorly defined area on the left (hollow arrow) corresponds to the biopsied region. **b**. Axial contrast-enhanced FS T1W MR image of the same patient as in a. The left orbital lesion shows diffuse post-contrast enhancement (asterisk). Enhancing soft tissue is seen extending along the left superior orbital fissure into the left cavernous sinus (arrow) and the dura along the left greater wing of sphenoid. **c.** Axial contrast-enhanced FS T1W image at the level of the maxillary sinus demonstrates perineural spread along the pterygopalatine fossa and maxillary nerve (arrows). Hollow arrows in b and c point at extra-orbital involement. **d.** ADC map reveals restricted diffusion of the orbital lesion with very low ADC values (ADC = 0.7 × 10 ^−3^ mm^2^/s) suggesting lymphoma. The optic nerve shows even lower ADC values (thin arrows) due to compression and ischemia. **e.** Colour coded DTI map shows major reduction of FA values in the left optic nerve (thin arrows). FA values were 0.4–0.5 on the left and 0.56–0.58 on the right. **f.** FDG PET/CT demonstrates high tracer uptake in the orbit (asterisk), along the superior orbital fissure and in the cavernous sinus and sphenoid (arrows) confirming findings revealed in b. SUVmean = 10, max = 16. Other hypermetabolic lesions were found in the neck nodes, mediastinum, and abdomen. Biopsy of orbital contents revealed NHL

NECT typically shows a hyperdense mass involving the lacrimal gland. The tumour usually molds to encase surrounding orbital structures. Significant enhancement may be seen on CECT. Bony destruction or perineural spread suggests an aggressive histology. High-cellularity tumours appear moderately hypointense on T1W and T2W MR images (Figs. 13 and 14). CEMRI usually shows avid enhancement (Fig. 14). At times, isolated involvement of the extraocular muscles or diffuse ill-defined orbital infiltration may be seen (Fig. 14) [1, 4, 66, 67].

Other infiltrative T2 moderately hypointense lesions namely benign orbital lympoproliferative disorders (OLPD), inflammatory orbital pseudotumour (IOP), and granulomatous diseases such as sarcoidosis and metastases (described subsequently) are common imaging differentials [1, 9, 68, 69].

Orbital lymphomas typically show very low ADC values, usually between 0.44-0.92 × 10 ^−3^ mm^2^/s (Figs. 13 and 14). Studies have proven that orbital lymphoma can be differentiated from IOP (ADC range 1.02 - 2.28 × 10 ^−3^ mm^2^/s) with 100 % accuracy using an ADC threshold of 1.0 × 10^−3^mm^2^/s and an ADC ratio of less than 1.2 × 10^−3^mm^2^/s [9–12, 68]. Similarly, lymphomas show significantly lower ADC values as compared to Ig-G4 related disease (mean ADC 1.67 × 10 ^−3^ mm^2^/s) [69] as well as metastases (ADC range 0.9-1.6 × 10 ^−3^ mm^2^/s) [9, 10, 12]. In a study by Haradome et al, the mean ADC and contrast-enhancement ratio of orbital lymphomas was found to be significantly lower than that of benign OLPD, thereby aiding in their differential diagnosis [70].

Sarcoidosis may mimic lymphoma at clinical presentation and at cross-sectional imaging. Sarcoidosis is a chronic, multisystemic, granulomatous disorder which commonly involves the orbit. It can cause mass-like lacrimal gland and muscle infiltration (often bilateral), optic nerve thickening and enhancement or pseudotumoural intra-orbital masses. There may be associated intracranial extension. The imaging findings may be exactly similar to lymphoma, even on DWI [12]. Bilateral hilar adenopathy, lung lesions, and elevated serum angiotensin-converting enzyme levels point to sarcoidosis. Often, a biopsy is required to demonstrate the non-caseating granulomas of sarcoidosis.

Lymphomas commonly show high FDG uptake (Figs. 13 and 14). Whole body PET CT has become part of standard staging of orbital lymphomas and is probably used more widely for this disease than any other orbital tumour Although, low-grade MALT lymphomas may show relatively low FDG uptake, PET CT has shown very good results in the detection of systemic metastases of even low-grade orbital lymphomas, which were not detected by conventional imaging [18, 20, 71, 72]. MRI PET holds promise in evaluating post-treatment response of lymphomas, especially in children [22, 73].

### Bone and sinus compartment

#### Fibrous dysplasia (FD)

FD is a developmental disorder of the bone in which normal bone marrow is replaced by fibro-osseous tissue with expansion of the medullary cavity. Cranio-facial fibrous dysplasia is commonly involves the frontal, ethmoid or sphenoid bones. It is typically seen in children and adolescents with slight female predilection. FD is monoostotic in 70-80 % cases and polyostotic in the rest. Orbital bony involvement leads to hypertelorism, exophthalmos, visual impairment and blindness. Surgery is needed to correct facial deformity or severe optic nerve compression [1, 26, 51].

FD shows replacement of lamellar bone with abnormal metaplastic immature woven bone, forming irregular curvilinear trabeculae on histology; this appearance has been described as “Chinese characters” or alphabet soup. The fibrous portion contains bland spindle cells without significant mitotic activity. Haemorrhage and cystic changes may be seen [26, 51].

**Fig. 15:**
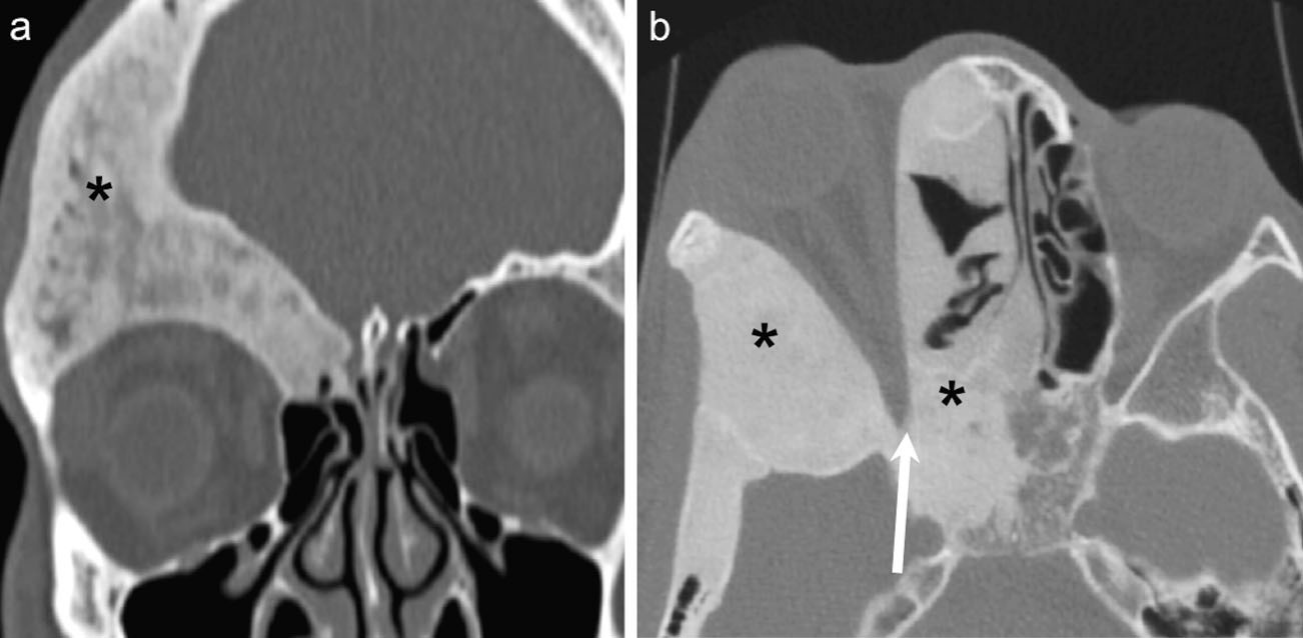
Two different patients with FD involving the facial and orbital bones. Characteristic ground-glass appearance (asterisks) and expansion of the medullary cavity is seen in both cases on NECT. In **b**, bony expansion of the right sphenoid wing and of the right anterior clinoid process causes severe narrowing of the right optic canal (arrow), thereby requiring decompressive surgery

**Fig. 16:**
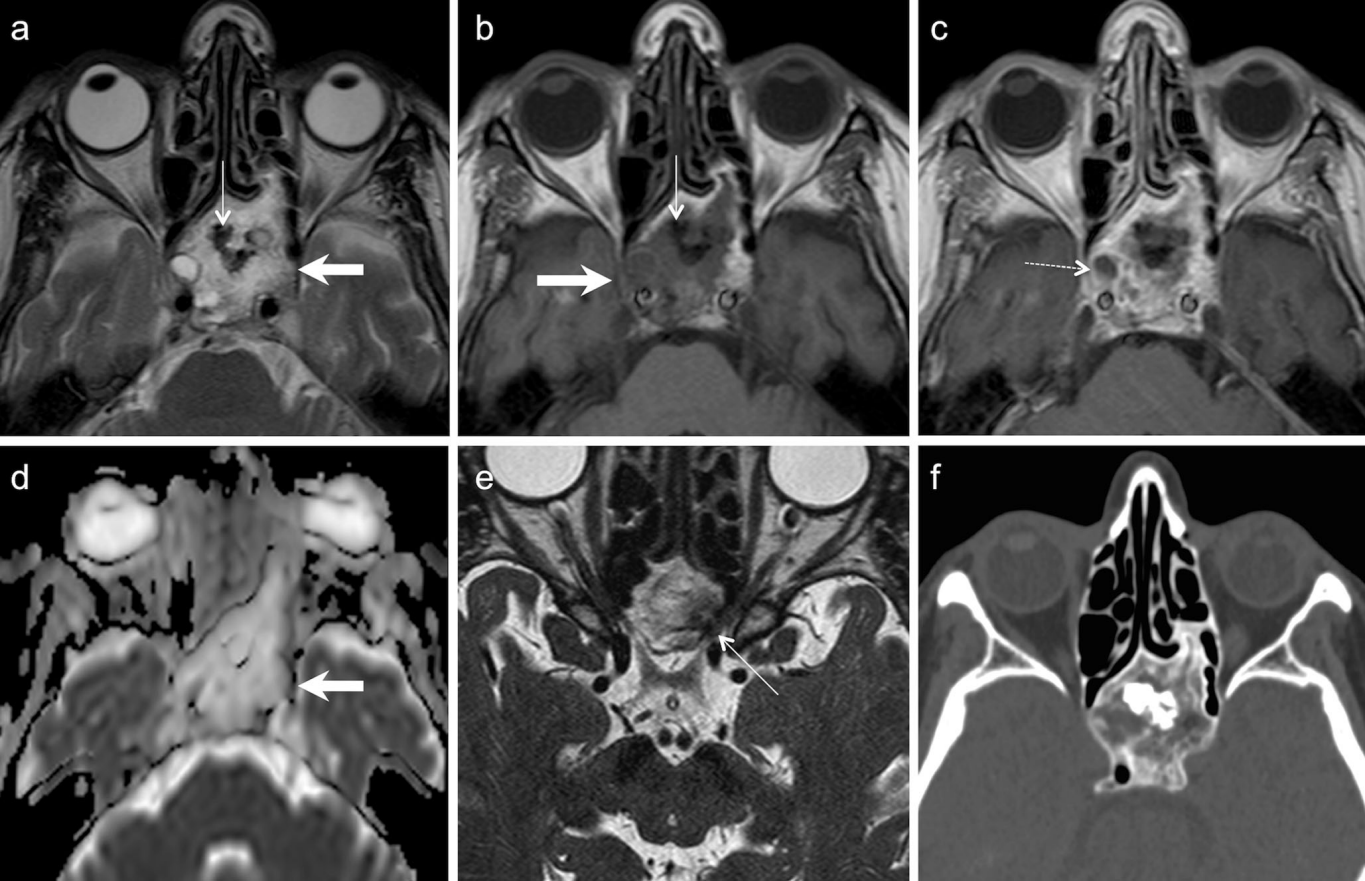
64-year-old female patient with headache and left vision loss underwent MRI. **a.** Axial T2W image of the orbits shows deformity and expansion of the sphenoid body. The medullary cavity is replaced by tissue with mixed hyperintense (thick arrow)–hypointense (thin arrow) signal. **b**. Axial T1W image of the same patient shows the lesion to be mainly isointense to brain parenchyma (thick arrow) and with strongly hypointense central areas (thin arrow). On the left, there are hyperintense peripheral regions. **c.** Axial contrast-enhanced T1W image shows inhomogeneous contrast enhancement (thick arrow) and cystic portions (dashed arrow). **d.** ADC map reveals increased diffusion (ADC = 2.3 × 10 ^−3^ mm^2^/s) suggesting a benign lesion (thick arrow). FD was suspected. **e.** Oblique reformatted axial image from 3D T2W sequence shows compression of the left optic nerve (thin arrow) at the level of the optic canal. **f.** Axial NECT image shows ground-glass appearance and irregular ossification of the involved bones. Part of the lesion was resected to decompress the right optic nerve. Histology revealed psammomatous variant of FD

Radiographs and CT shows bony expansion with “ground glass” appearance (Fig. 15). There may be consequent narrowing of the optic nerve canal and impingement of the optic nerve (Figs. 15 and 16). The fibrous stroma and osteoid material typically appears hypointense on T1W images (Fig. 16). On T2W images the signal intensity may be low (18-38 % cases), intermediate (18 %) or high (62–64 %) (Fig. 16). Marked enhancement may be seen on CEMRI [1, 51] (Fig. 16), as well as fluid-fluid levels. Similar to benign lesions elsewhere in the head and neck, FD shows significantly higher ADC values compared to malignant bone tumours [74, 75] (Fig. 16).

FD can appear hypermetabolic on PET CT with high SUV values thereby simulating malignancy [76, 77]. Nevertheless, the high ADC values and the characteristic CT aspect help to avoid this pitfall.

### Multi-compartmental tumours

#### Venolymphatic malformation (VLM)

VLM, also called lymphangioma, is a congential, hamartomous vascular malformation with variable lymphoid and venous vascular elements. It is hemodynamically isolated from systemic drainage. VLM accounts for 5 % of paediatric orbital tumours; about 60 % cases are diagnosed by 16-20 years of age. It is commonly extraconal; however, it can be intraconal or multicompartmental. Proptosis, diplopia, and optic neuropathy are common presenting symptoms. Sudden increase in proptosis indicates haemorrhage within the lesion. Unlike capillary hemangiomas, which involute over time, VLM grow with the patient, especially in puberty. Treatment is usually conservative. Percutaneous sclerotherapy is performed in select cases. Surgery is difficult due to the transpatial nature and also due to risk of recurrence [2, 4, 26, 45, 60].

**Fig. 17:**
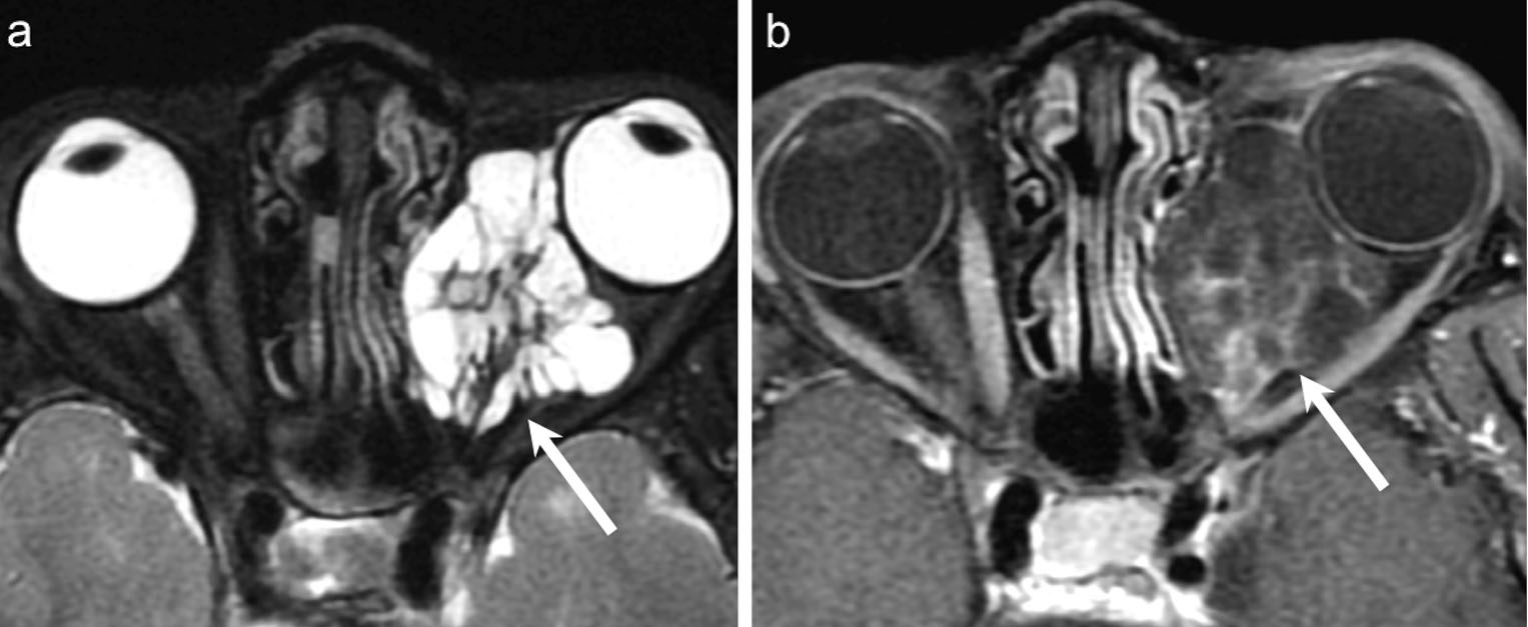
15-year-old girl with a left orbital VLM. **a.** Axial FS T2W image shows a mutliloculated cystic lesion involving the intraconal and extraconal compartments of the left orbit (arrow). The strongly hypointense septae of the VLM show hemosiderin staining. **b.** Contrast-enhanced FS T1W MR image of the same patient shows minimal enhancement (arrow) along the intervening septae of the VLM

VLM typically contain irregular shaped venous and lymphatic channels lines by flattened endothelial cells and interspersed connective tissue. The cystic spaces may show blood products or lymphatic fluid within [26, 45, 60]. VLM are usually seen on CT and MRI as poorly circumscribed, lobulated, transpatial lesions. Hemorrhagic and proteinaceous contents may appear hyperdense on NECT. Fluid-fluid levels are commonly seen. Calcification and bony erosions are uncommon. The lesion shows variable signal on T1W and T2W MR images (Fig. 17). As against capillary hemangiomas, flow voids are absent. Fat-saturated CEMRI images are ideal for mapping the lesion. Rim enhancement is commonly seen (Fig. 17) [2, 4, 26, 45, 60]. VLM show increased diffusion [13]. Their ADC values have been reported in the range of 1.44–1.53 × 10 ^−3^ mm^2^/s and 1.75-2.2 × 10 ^−3^ mm^2^/s [9, 12]. On FDG PET CT, there is no focal uptake.

#### Orbital plexiform neurofibroma (OPNF)

OPNF is a hamartoma of neuroectodermal origin accounting for 1-2 % of all orbital tumours, typically arising in the first decade of life. OPNF is diagnostic of NF-1. OPNF can involve any peripheral nerve, but the sensory nerves of the orbit are commonly involved. It is usually associated with other findings of NF-1 such as ONG, sphenoid wing dysplasia, and buphthalmos. OPNF usually presents with nodular periorbital masses, loss of vision, and proptosis. The infiltrative serpentine masses extend in both the intraconal and extraconal compartments. Progressive glaucoma, optic nerve atrophy, and blindness are eventual complications. The risk of malignant sarcomatous degeneration is about 10 %. ONSF are usually not amenable to surgery; however, debulking may be necessary for preservation of vision or cosmetics [4, 26, 78].

**Fig. 18:**
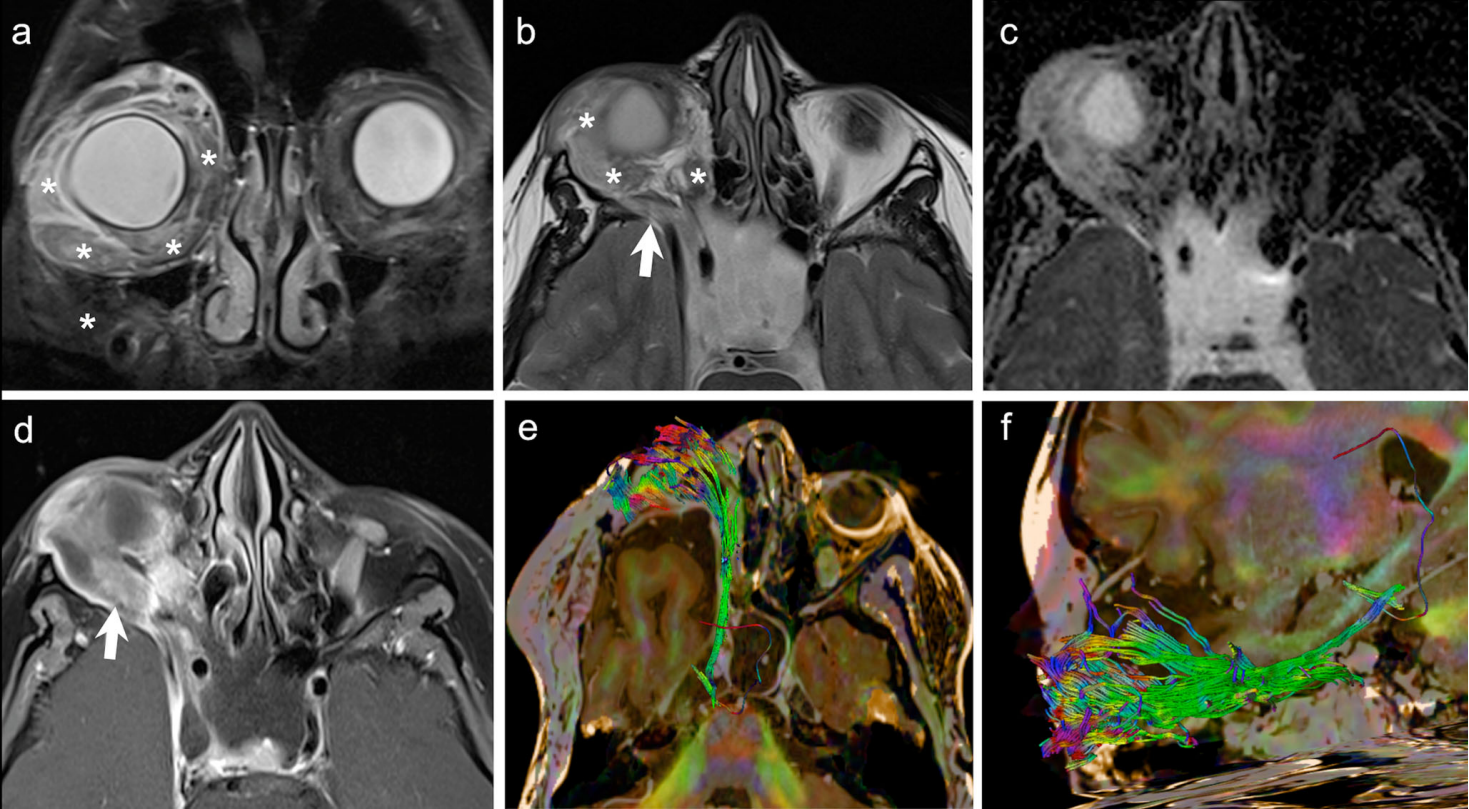
28-year-old female patient with NF-1. MRI was performed for pre-surgical planning. Coronal STIR (**a**) and axial T2W (**b**) images show poorly circumscribed, serpentine masses (asterisks) within the right orbital conal-extraconal and preseptal compartments. Findings are typical of an OPNF. There is associated dural ectasia of the right optic nerve sheath. Classic right-sided sphenoid wing dysplasia is also noted (arrow). The ADC map (**c**) shows no restriction of diffusion (ADC = 1.3 × 10^−3^ mm^2^/s) in keeping with the benign histology. **d.** Axial contrast-enhanced FS T1W image shows heterogeneous strong contrast enhancement of the OPNF (arrow). Axial (**e**) and sagittal (**f**) DTI 3D tractography views show complete disorganization of fibres within the OPNF surrounding the globe and optic nerve

Unlike schwannomas, it is not possible to distinguish host nerve fibres separately from OPNF. The host nerve fascicles are irregularly expanded by myxoid accumulation, tumourous Schwann cells, fibroblasts, and collagen fibres [26]. Plain radiography may detect a classical defect in the greater wing of sphenoid called “Harlequin eye” appearance. Both CT and MRI show the characteristic orbital and periorbital infiltrative soft tissue masses, associated OPNF, and sphenoid wing dysplasia (Fig. 18). On T2W MRI, the nodular masses typically appear hyperintense with central low signal called the “target sign”. The nodular masses show variable post contrast enhancement (Fig. 18). MRI is ideal for evaluation of the associated intracranial abnormalities. Infantile/capillary hemangioma, VLM, and RMS may mimic OPNF on imaging; however, the other associated stigmata of NFI are absent in these cases [4, 78].

OPNF show mixed or increased diffusion [13]. DTI with tractography reconstruction is increasingly being used for the pre-operative mapping of neurogenic tumours originating in the head and neck (typically from the brachial plexus), such as schwannomas and neurofibromas [79–81]. DTI with tractography reconstruction can accurately detect alterations of involved nerve fascicles, such as displacement, stretching, bowing, or rupture (Fig. 18). FDG-PET CT and more recently MRI PET are sensitive and specific tools to detect sarcomatous transformation in OPNF [82, 83].

#### Inflammatory orbital pseudotumour (IOP)

IOP is the most common cause of a painful orbital mass in adults and the third most common orbital disease after thyroid ophthalmopathy and lymphoproliferative disorders. IOP is a benign, non-infective, inflammatory condition without identifiable local or systemic causes and is typically seen in the 4th-6th decade. The disease may present as scleritis, uveitis, lacrimal adenitis, myositis, perineuritis, or diffuse orbital inflammation. The classic clinical triad consists of unilateral orbital pain, proptosis, and impaired ocular movement. There may be associated fibrosing mediastinitis or retroperitoneal fibrosis. The diagnosis of IOP is made after exclusion of other pathologies like thyroid ophthalmopathy, lymphoma, Wegener’s granulomatosis, and sarcoidosis. Treatment with steroids shows dramatic response, which helps to confirm the diagnosis [1, 2, 68, 84, 85].

**Fig. 19:**
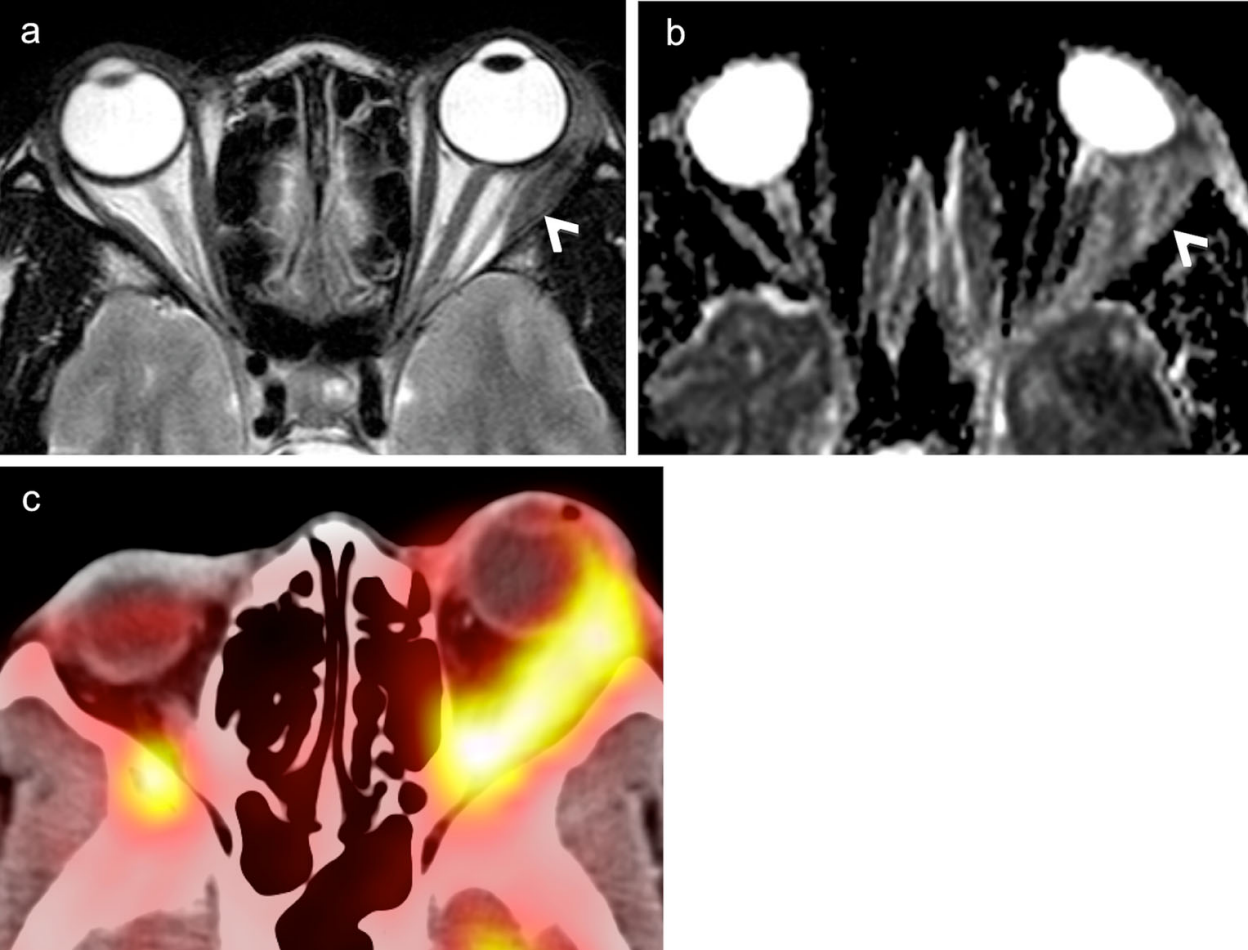
35-year-old female patient with left histologically proven orbital IOP. **a.** Axial T2W image shows a plaque-like hypointense extraconal lesion (arrowhead), adjacent to the lateral rectus muscle. Lymphoma was considered as an imaging differential. **b.** ADC map of the orbits of the same patient shows an ADC value of 1.1 × 10^−3^ mm^2^/s (arrowhead), which is higher than the ADC value expected for lymphoma. **c**. Axial PET/CT image of the same patient shows high FDG uptake within the lesion (arrow) (SUVmean = 5, SUVmax = 6) mimicking lymphoma

IOP is characterized histologically by a mixed inflammatory infiltrate, which consists of lymphocytes, plasma cells, macrophages, and eosinophilia. Fibrosis may also be seen [84, 85]. Imaging findings may be non-specific. CT and MRI may show mass-like enhancing soft tissue within the orbit, streaky fat stranding, lacrimal gland enhancement, and optic nerve sheath enhancement (Fig. 19). The extraocular muscles are commonly enlarged and show enhancement. These inflammatory masses are usually hypointense to fat on T1W images and iso-hypointense on T2W images (Fig. 19). They can extend to involve the pterygopalatine fossa, nasopharynx, and cavernous sinus [1, 2, 68, 84, 85].

IOP is a great imaging mimic of several other pathologies. These include other causes of myositis (thyroid ophthalmopathy, infective cellulitis) and other T2-hypointense infiltrative lesions (lymphoma, IgG4-RD, and granulomatous diseases). Thyroid ophthalmopathy causes bilateral inflammation of the extra-ocular muscles, commonly involves the inferior and medial rectus muscles, often spares the myo-tendinous junctions, and is associated with elevated thyroid-stimulating hormone levels. IgG4-RD can show similar features as thyroid ophthalmopathy, however, with normal thyroid stimulating hormone levels. Infective cellulitis is commonly associated with fever, leukocytosis, and abscess formation. Clinical features such as pain, conjunctival congestion, and eyelid oedema help to distinguish IOP from lymphoma, which commonly presents with a painless palpable mass [1, 2, 68, 84–88]. As described previously, ADC values help to accurately differentiate between IOP and lymphoma (Fig. 19) [9–12, 68]. IOP has been reported to show very high FDG uptake mimicking malignancy (Fig. 19) [89, 90].

#### IgG4-RD

Immunoglobulin G4-related disease (IgG4-RD) is a chronic, systemic autoimmune, fibro-inflammatory condition. Its most common manifestation is autoimmune pancreatitis; however, retroperitoneal fibrosis, sclerosing cholangitis, interstitial nephritis, periarteritis, Riedel’s thyroiditis, chronic dacryoadenitis, Mikulicz disease, and certain orbital inflammatory pseudotumours may frequently be encountered. As with IOP, IgG4-RD shows a dramatic response to corticosteroid therapy, although spontaneous resolution has been described [85–88]. Practically any part of the orbit can be involved, and the typical histological features, regardless of the affected site, are dense lympho-plasmacytic infiltration with abundant IgG4-positive plasma cells, storiform-type fibrosis with obliterative phlebitis [86–88].

**Fig. 20:**
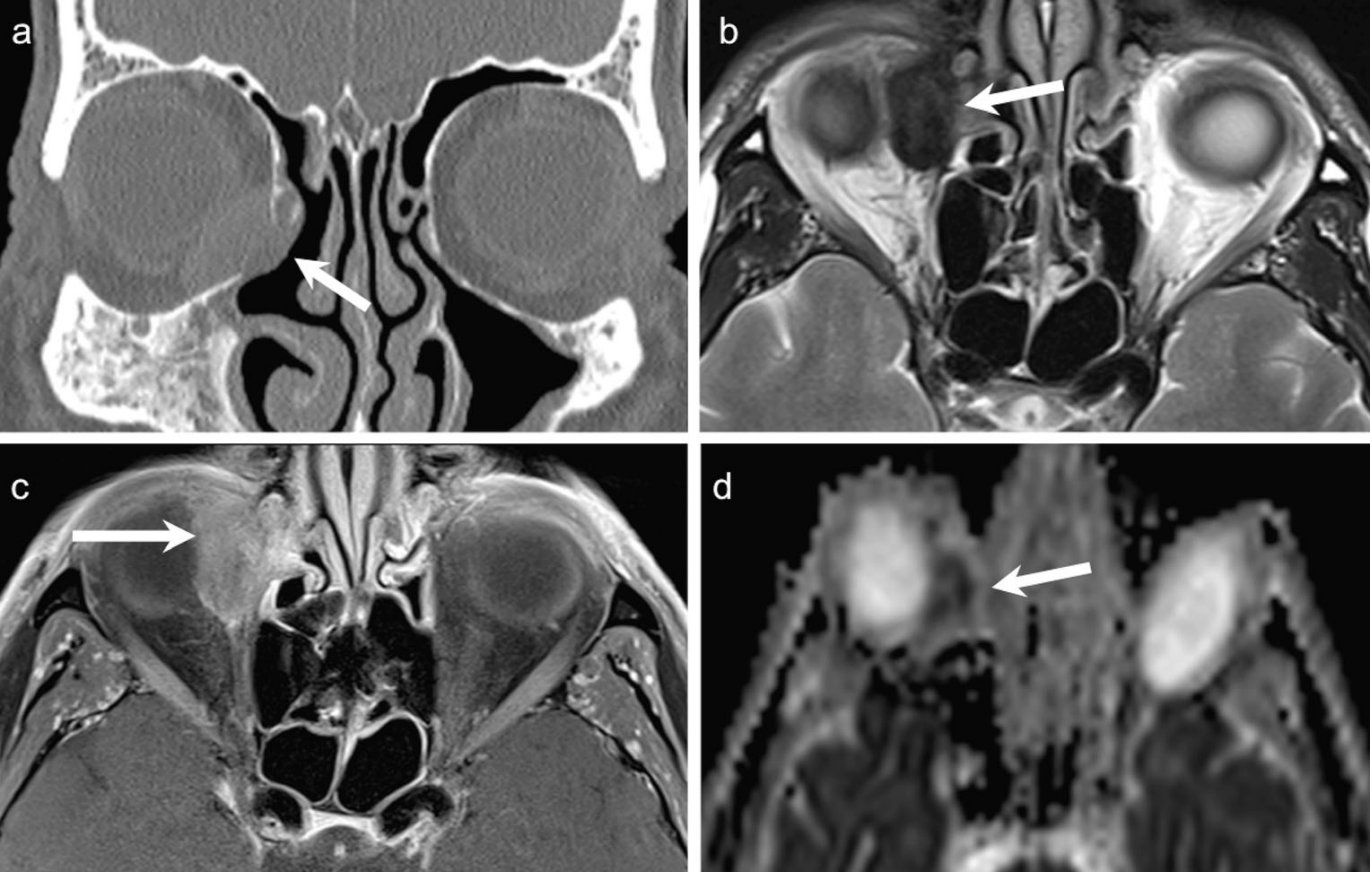
50-year-old male patient with biopsy-proven IgG4-RD of the right orbit. Coronal bone-window CT image of the orbits (**a**) shows a well-circumscribed, extraconal, soft tissue-density mass located inferomedially in the right orbit. It is associated with bony erosion of the lamina papyracea (arrow). **b.** Axial T2W image of the same patient shows very low signal within the well-circumscribed lesion (arrow). Note that it is lower than usually seen in lymphoma. **c.** Axial contrast-enhanced FS TIW image shows homogeneous non-specific enhancement within the lesion (arrow)**. d.** ADC map shows restricted diffusion (ADC = 0.9 × 10 ^−3^ mm^2^/s)

On MRI, IgG4-RD typically demonstrates significant hypointensity on T1 and T2W images with marked enhancement after contrast administration. Lymphoma shows similar MRI features on conventional sequences (Fig. 20) [86–88]. As described previously, DWI with ADC mapping may help to differentiate between the two pathologies (Fig. 20) [69]. PET CT can help to detect extra-pancreatic involvement in IgG4-RD [91].

#### Metastases

Metastases represent the most common orbital malignancy. Common sources for orbital metastases include melanomas, breast and lung cancers in adults, and neuroblastomas in children. Metastases commonly involve the orbital bones and extraconal compartment; however, they may also involve the choroid. Painful proptosis is the most common presentation, the exception being cirrhotic breast carcinoma metastases, where enophthalmos is a typical finding due to rectus muscle infiltration and contraction [1, 2, 4].

**Fig. 21:**
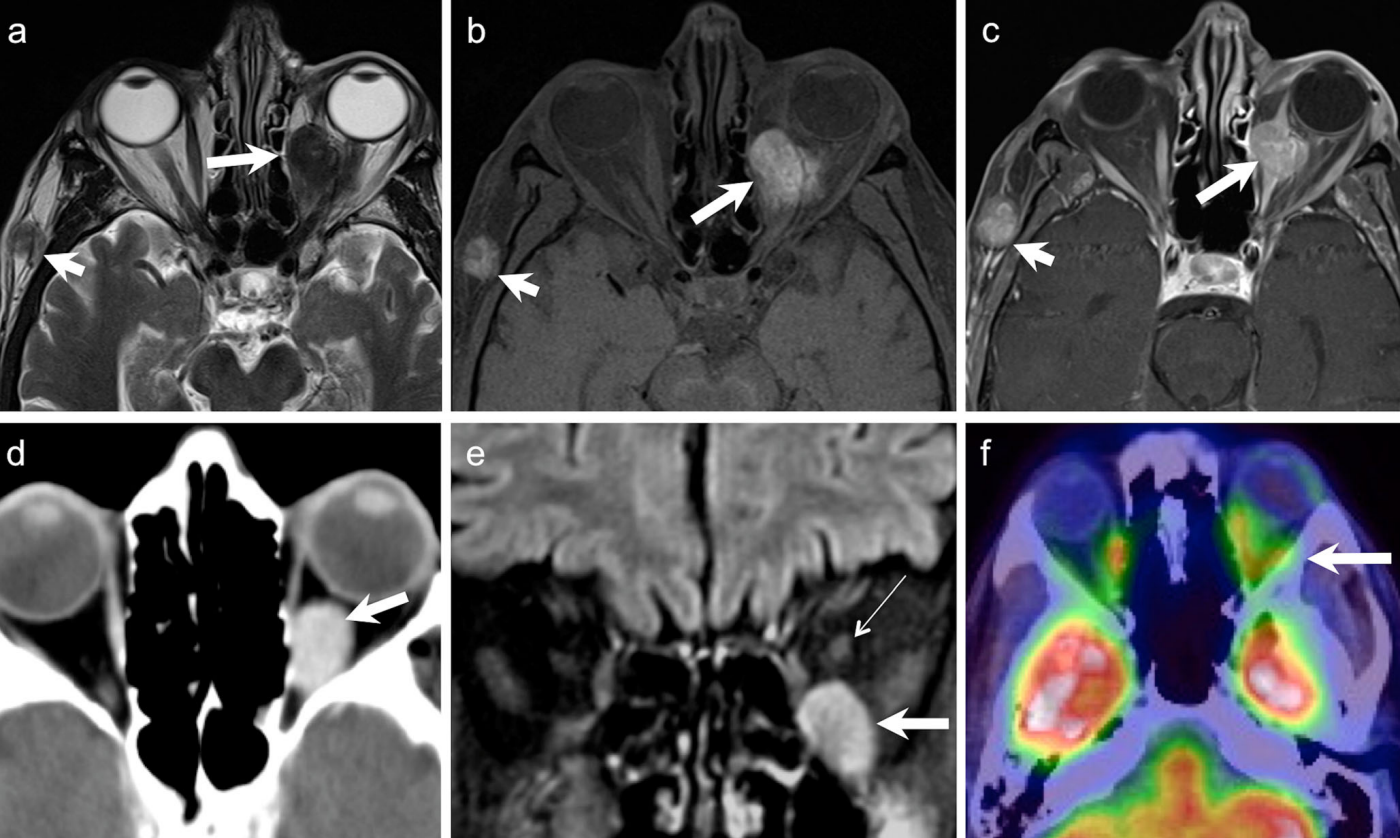
Two different patients with orbital metastases. **a – c.** 74-year-old female patient with diplopia and a history of melanoma of the scalp 7 years previously. T2W (**a**), unenhanced FS T1W (**b**) and contrast-enhanced FS T1W (**c**) images of the orbits shows a conal-extraconal mass in the left orbit (arrows) and a second mass with similar imaging features in the suprazygomatic right masticator space (short arrows). The strongly hyperintense signal in b (arrows) suggests the presence of haemorrhage and/or melanin. Imaging findings are strongly suggestive of metastases from melanoma. Findings were confirmed by biopsy. **d – f.** 71-year-old female patient with known breast cancer. **d.** Axial CECT image shows a well-circumscribed enhancing mass in the left orbit in extraconal location (arrow). **e.** Coronal STIR image of the same patient shows non-specific moderately high signal of the metastatic deposit (thick arrow). Thin arrow points to the left optic nerve. **f.** Axial PET/CT image of the same patient shows physiological high FDG uptake in the extra-ocular muscles making it difficult to detect the metastatic lesion (arrow). As the imaging findings in this case are non-specific, the diagnosis of orbital metastasis can be suggested only when the clinical background is known. Biopsy confirmed metastasis from breast cancer

Orbital metastases show histology typical to the primary tumour. High mitoses and necrosis may be seen (Fig. 21). When the primary tumour is unknown, immunohistochemistry may aid in diagnosis.

CT and MRI may show an enhancing infiltrative extraconal mass (Fig. 21). There can be associated bony destruction. Choroidal metastases from melanoma may appear hyperdense on NECT and hyperintense on T1W MR images. CEMRI also helps to detect associated intracranial metastases [1, 2, 4]. As described previously, metastases can mimic lymphomas on conventional CT/MR sequences. DWI can help to differentiate between the two [9, 10, 12]. An orbital metastatic deposit may be incidentally diagnosed for the first time on a PET CT. PET CT can also help to detect the primary tumour site and metastatic deposits to other organs [17–19].

#### Lid tumours with orbital extension

**Fig. 22:**
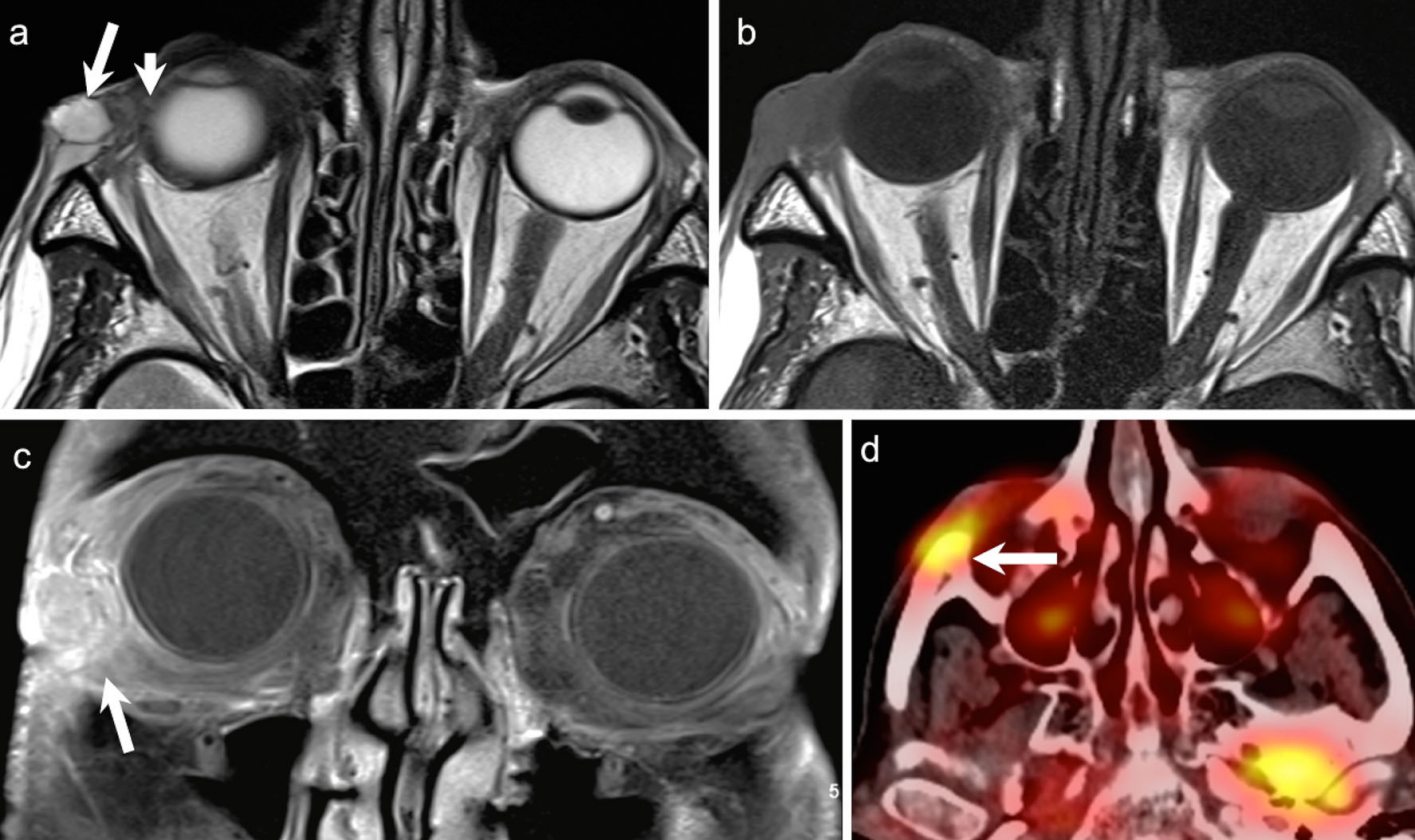
60-year-old male patient with desmoplastic melanoma of the right eyelid. **a.** Axial T2W image shows a heterogeneous signal-intensity lesion involving the subcutaneous tissue of the right lateral canthus (big arrow) with an infiltrating component extending into the extraconal compartment of the right orbit. There is suspicious invasion of the muscle cone and the globe (short arrow). **b:** Axial T1W image of the same patient does not show hyperintense signal within the lesion in keeping with low melanin content. **c.** Coronal contrast-enhanced FS TIW image reveals avid enhancement within the lesion. **d.** Axial PET/CT image of the same patient shows high FDG uptake within the lesion due to high glucose metabolism. No other lesions were detected in the rest of the body

Common malignant tumours of the eyelid include squamous cell carcinoma (SCC), basal cell carcinoma, melanoma, sebaceous cell carcinoma, and lymphoma. These tumours are often highly aggressive and show contiguous invasion of orbital structures. About 43 % of SCC of the eyelid show orbital involvement (Fig. 22). Perineural spread along the branches of the trigeminal nerve is also common. Orbital exenteration is required for controlling local disease spread which may be relentless and potentially fatal. Cross-sectional imaging helps in the local staging of the disease [18, 92, 93]. PET CT plays a valuable role in detecting local nodal and distant metastases from eyelid carcinomas [18, 59].

## Conclusion

Evaluation of orbital masses requires a multimodality approach to balance the strength and weaknesses of each modality. Combining accurate clinical information with appropriate imaging modalities and being aware of potential diagnostic pearls and pitfalls helps to obtain the best results. Future advances in functional imaging are likely to make a significant impact on ophthalmological cancer imaging. We hope that this review has satisfactorily served the purpose of highlighting these points.

## Abbreviations

ACC: Adenoid cystic carcinoma
ADC: Apparent diffusion coefficient
BMT: Benign mixed tumour
CECT: Contrast-enhanced CT
CEMRI: Contrast-enhanced MRI
DTI: Diffusion tensor imaging
DWI: Diffusion-weighted imaging
FA: Fractional anisotropy
FD: Fibrous dysplasia
FDG: Fluoro-2-deoxy-D-glucose
PET CT: positron emission tomography CT
MRI PET: positron emission tomography MRI
FS: Fat saturated
FT: Full echo train
HR: High resolution
H&E stain: Haematoxylin-eosin stain
IHC: Immunohistochemistry
IgG4-RD: Immunoglobulin G4-related disease
IOP: Inflammatory orbital pseudotumour
MALT: Mucosa associated lymphoid tissue
NECT: Non contrast-enhanced CT
NF: Neurofibromatosis
NHL: Non-Hodgkin’s lymphoma
ONG: Optic nerve glioma
ONSM: Optic nerve sheath meningioma
OLPD: Orbital lympoproliferative disorders
OPNF: Orbital plexiform neurofibroma
RMS: Rhabomyosarcoma
RT: Radiotherapy
SCC: Squamous cell carcinoma
SUV: Standardised uptake value
US: Ultrasound
VLM: Veno-lymphatic malformation
